# Supplementary material for: Short-term acidification promotes diverse iron acquisition and conservation mechanisms in upwelling-associated phytoplankton
Source: Nat Commun. 2023 Nov 8;14:7215. doi: 10.1038/s41467-023-42949-1 (PMC10632500; doi:10.1038/s41467-023-42949-1)
Supplement: Supplementary file 1 — Supplementary Information [file 41467_2023_42949_MOESM1_ESM.pdf]

Short-term acidification promotes diverse iron acquisition and conservation mechanisms in upwelling-associated phytoplankton

Supplementary Information

Robert H. Lampe[1,2], Tyler H. Coale[1,2], Kiefer O. Forsch[3], Loay J. Jabre[4], Samuel Kekuewa[3], Erin M. Bertrand[4], Aleš Horák[5,6], Miroslav Oborník[5,6], Ariel J. Rabines[1,2], Elden Rowland[4], Hong Zheng[2], Andreas J. Andersson[3], Katherine A. Barbeau[3], Andrew E. Allen[1,2*]

[1]Integrative Oceanography Division, Scripps Institution of Oceanography, University of California San Diego, 9500 Gilman Drive, La Jolla, CA, 92093 USA
[2]Microbial and Environmental Genomics, J. Craig Venter Institute, 4120 Capricorn Lane, La Jolla, CA, 92037, USA
[3]Geosciences Research Division, Scripps Institution of Oceanography, University of California San Diego, 9500 Gilman Drive, La Jolla, CA, 92093 USA
[4]Department of Biology and Institute for Comparative Genomics, Dalhousie University, 1355 Oxford St, Halifax, Nova Scotia, Canada B3H 4R2
[5]Biology Centre, Institute of Parasitology, Academy of Sciences of the Czech Republic, 370 05 České Budějovice, CZ
[6]Faculty of Science, University of South Bohemia, 370 05 České Budějovice, CZ

***For correspondence:**
Dr. Andrew E. Allen
4120 Capricorn Lane
La Jolla, CA 92037
Email: aallen@jcvi.org
Phone: (858) 200-1826

This file includes:

Supplementary Figures 1-28
Supplementary Tables 1-7
References for Supplementary Material

Supplementary Figure 1. Temperature (°C, blue) and chlorophyll fluorescence voltages (V, green). from the CTD downcast prior to the start of the incubations. The horizontal dashed line indicates the depth of seawater collection for the ocean acidification experiments. The dotted line indicates the depth of collection for the *in situ* incubations. Data shallower than 24 m are not available for the cast for Experiment 2.

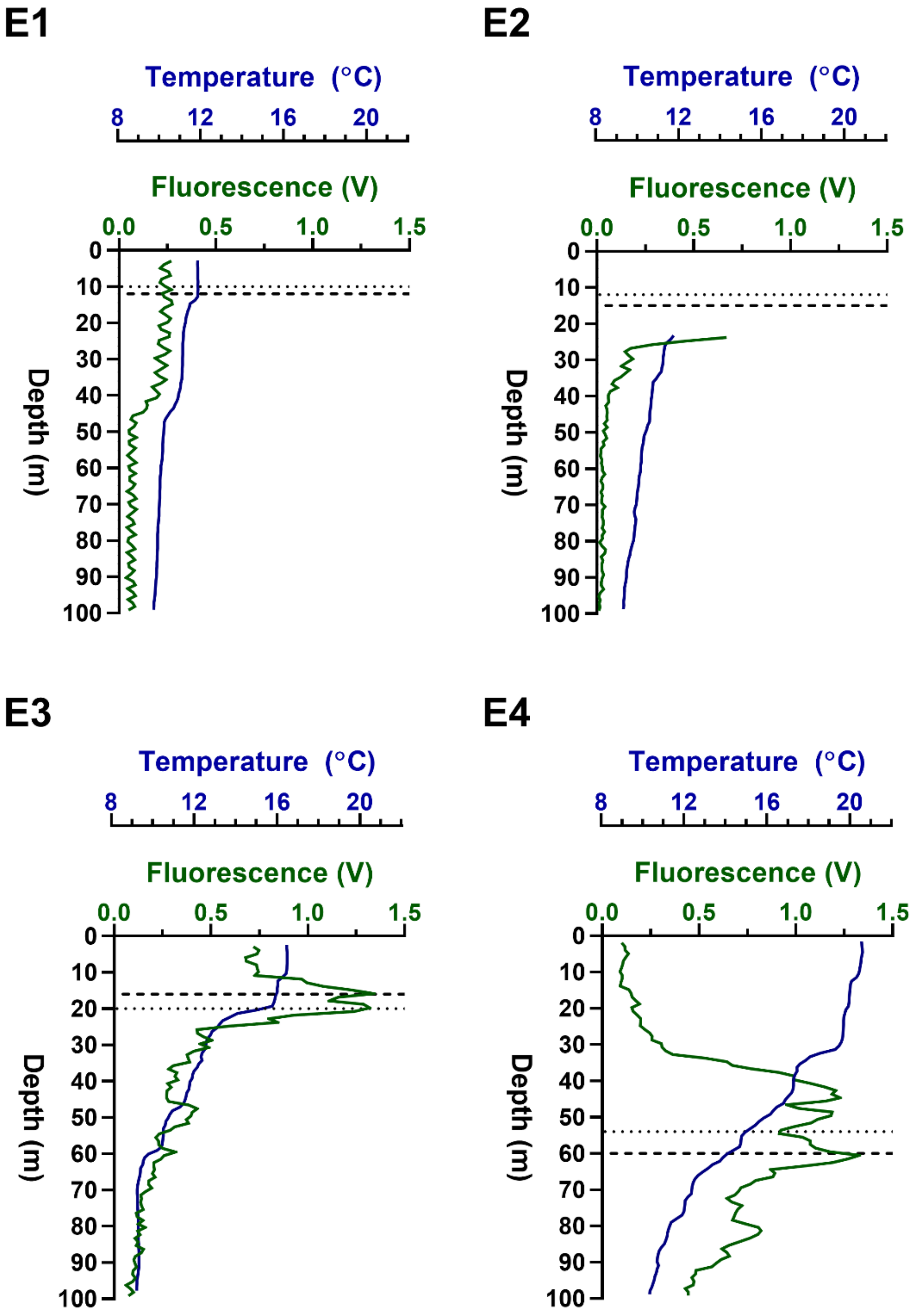

Supplementary Figure 2. pH (total scale, left y-axis) and carbonate ion concentrations [CO_3^{2-}] (µmol kg^{-1}, right y-axis) computed from dissolved inorganic carbon (DIC) and total alkalinity (TA) for each experiment (E). Timepoints (T) and CO_2 treatments (µatm) are denoted on the x-axis. Measured DIC and TA as well as calculated pH, pCO_2, and [CO_3^{2-}] are shown in Supplementary Table 3.

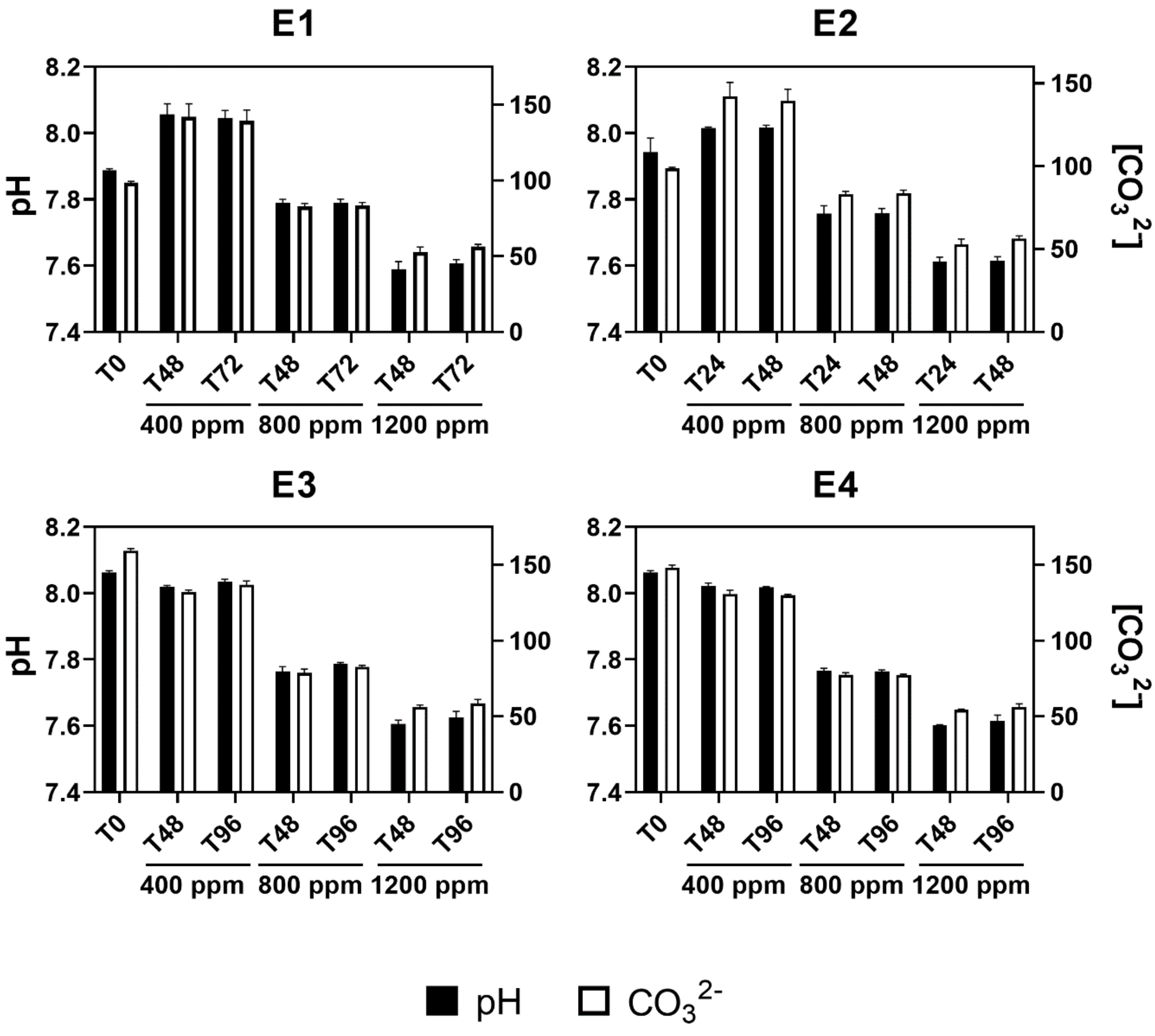

Supplementary Figure 3. Labile particulate iron concentrations (LpFe, nmol L^{-1}) calculated from total dissolvable iron minus dissolved iron concentrations. (a) Average concentrations from 10 to 25 m at locations near where the ocean acidification experiments were conducted (n = 1-2). Locations where seawater was collected for each experiment are numbered and shown as grey diamonds. (b, c) Depth profiles corresponding to the sites labelled b and c in panel a near the initial seawater collection for Experiment 4 at 60 m.

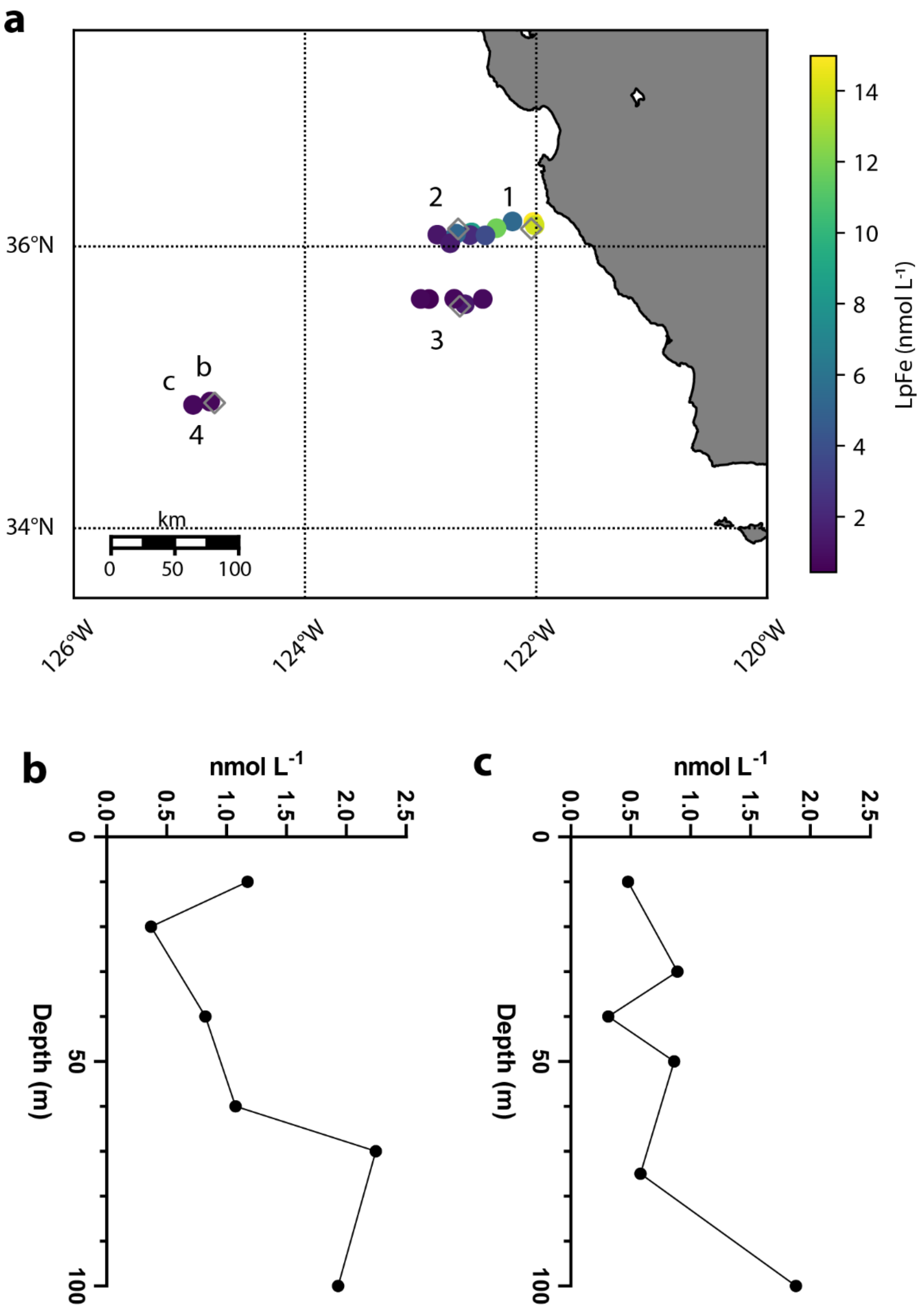

Supplementary Figure 4. Chlorophyll *a* concentrations (μg L^{-1}) from the parallel incubations.
(A, B, C, E) *In situ* experiments with control, iron addition (+Fe), or DFB addition (+DFB) treatments after 24 hours.
(D) Deckboard experiment during Experiment 3 with control and iron addition treatments sampled after 24 and 48 hours.
(F) *In situ* incubation initiated 24 hours after Experiment 4 with control, iron addition, nitrate addition, and iron plus nitrate addition treatments. Bottles were incubated for 48 hours.
Y-axis scales are different for each experiment. Letters denote significant differences between means where observed ($P < 0.05$, ANOVA with Tukey Test). Error bars represent the standard deviation of the mean ($n = 3$).

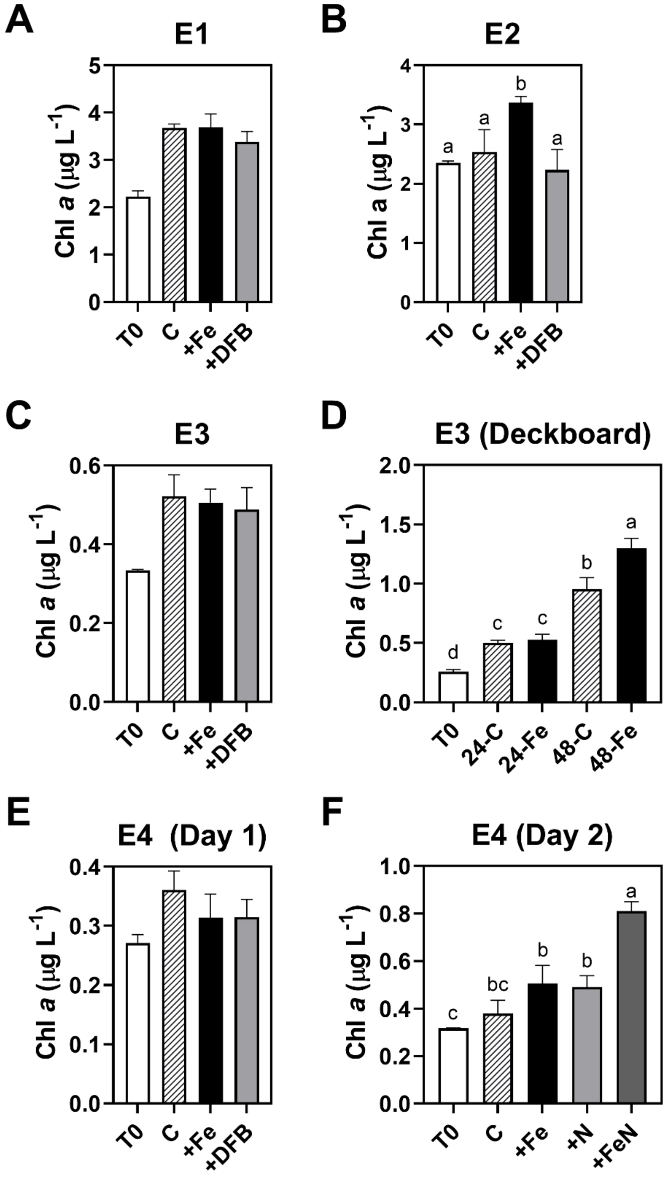

Supplementary Figure 5. Macronutrient concentrations (µmol L^{-1}) from the parallel *in situ* incubations at the initial time point (T0) and after 24 hours of incubation with control (C), iron addition (+Fe), or DFB addition (+DFB) treatments. Phosphate (PO_4) concentrations are one-tenth the plotted values. Y-axis scales are different for each experiment. Error bars represent the standard deviation of the mean (n = 3).

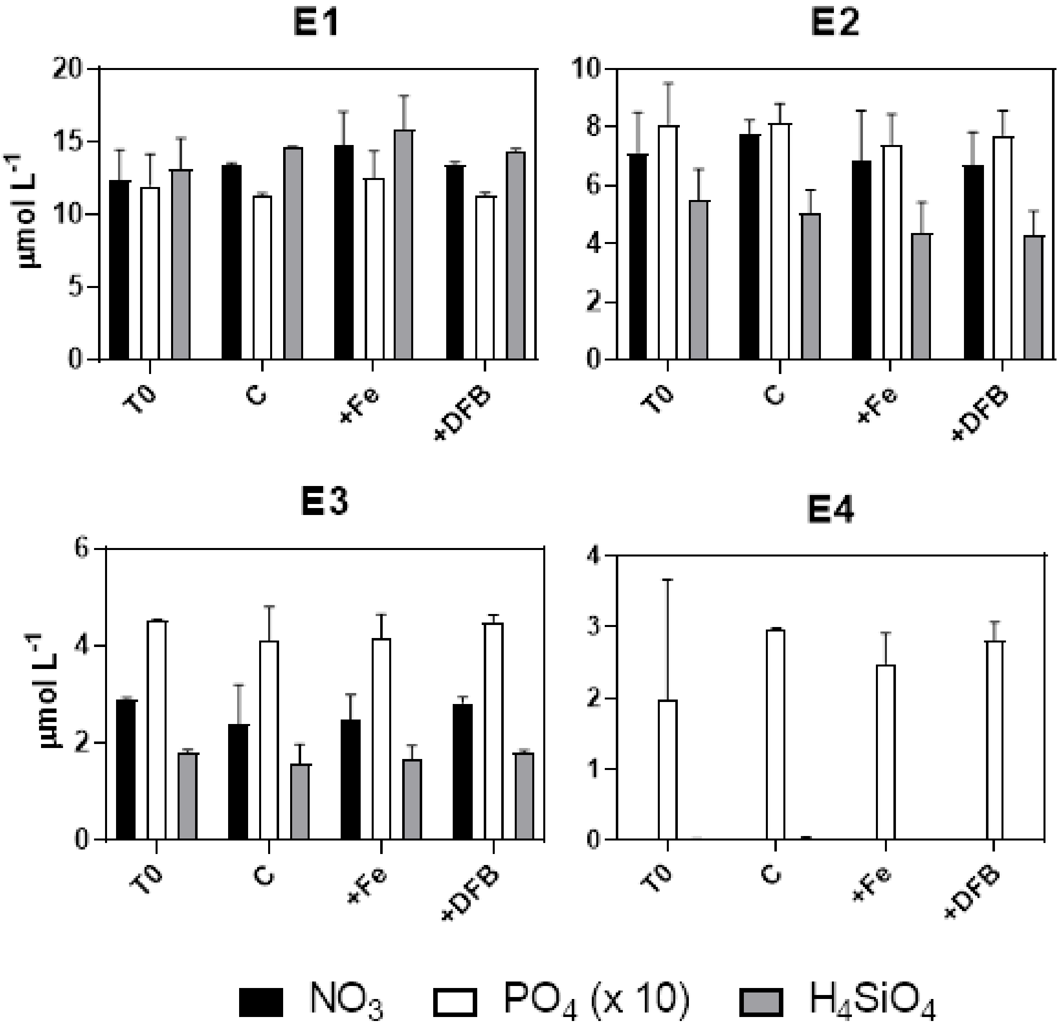

Supplementary Figure 6. Short-term iron-59 uptake rates normalized to particulate organic carbon (µmol Fe mol C^{-1} hr^{-1}) for inorganic iron ($FeCl_3$, black, left y-axis) and organically-complexed iron as ferrioxamine B (FeDFB, white, right y-axis). Error bars represent the standard deviation of the mean (n = 3 except the Experiment 4 800 ppm samples where n = 2).

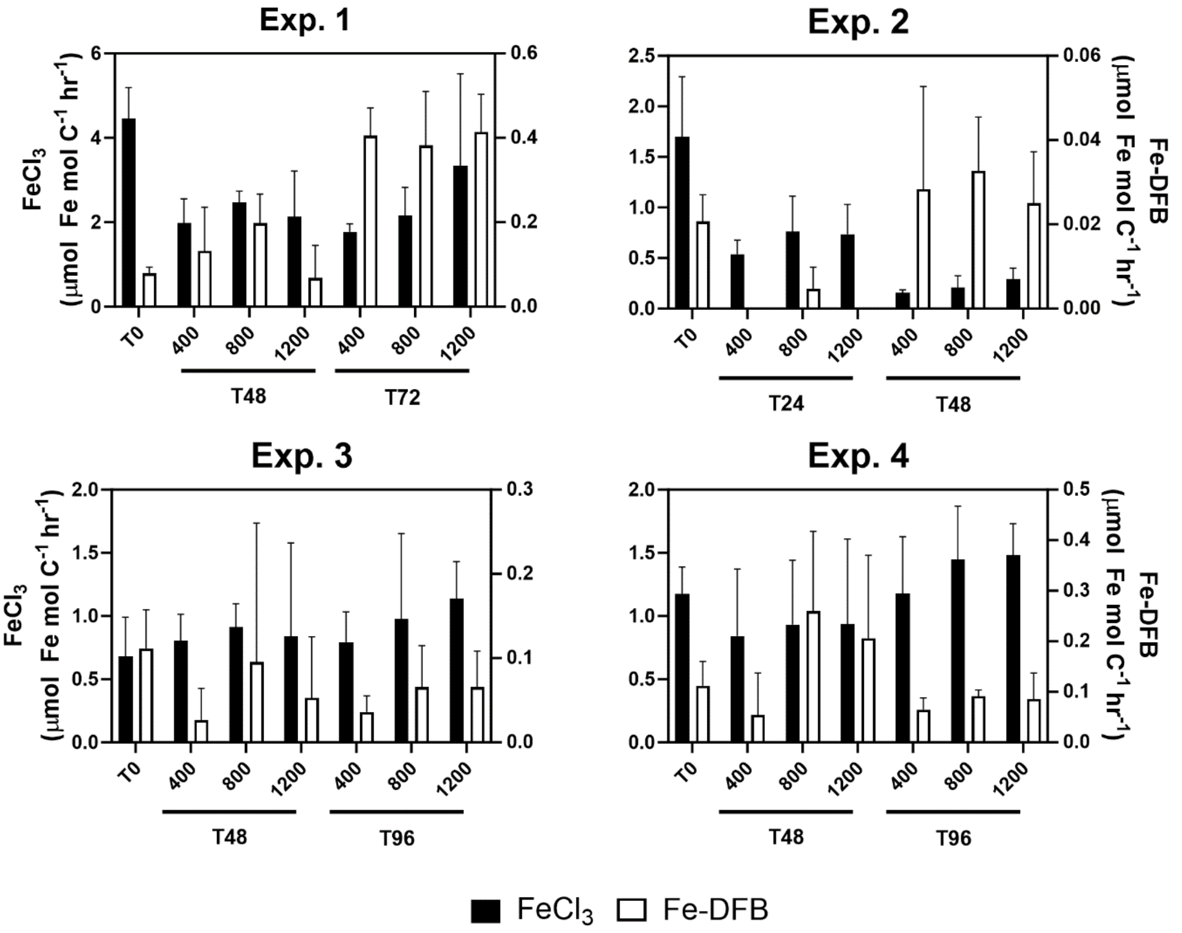

Supplementary Figure 7. Short-term iron-59 uptake rates over time
(A, B) normalized to chlorophyll *a* concentrations (pmol Fe µg Chl^{-1} hr^{-1})
(C, D) normalized to particulate organic carbon (µmol Fe mol C^{-1} hr^{-1})
(A, C) inorganic iron ($FeCl_3$) uptakes
(B, D) organically-complexed iron as ferrioxamine B (FeDFB) uptake rates
Error bars represent the standard deviation of the mean (*n* = 3 except the Experiment 4 800 ppm samples where *n* = 2).

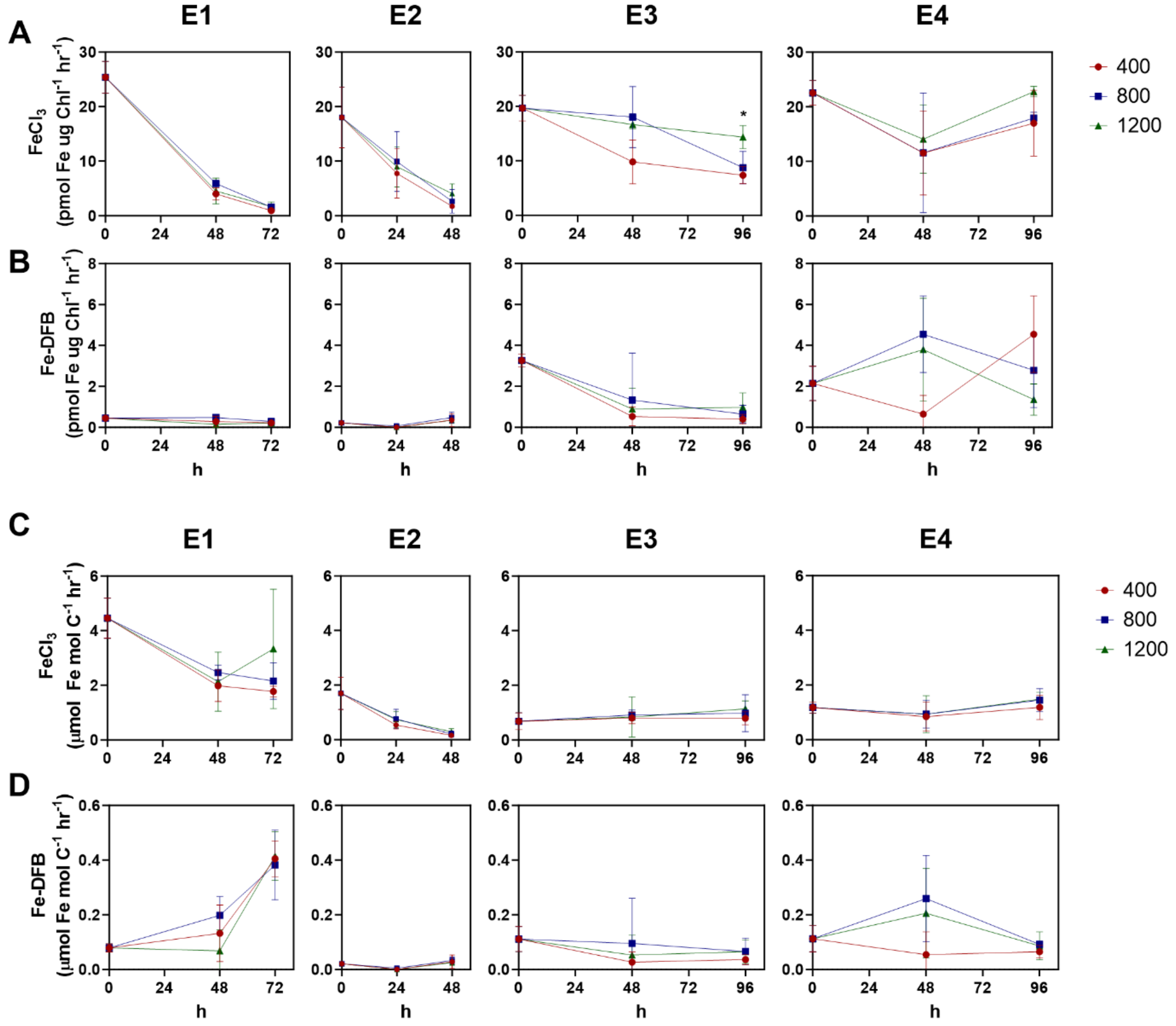

Supplementary Figure 8. Community growth rates (μ, d^{-1}) for each treatment within each experiment. Growth rates were not significantly different within each experiment ($P > 0.05$).

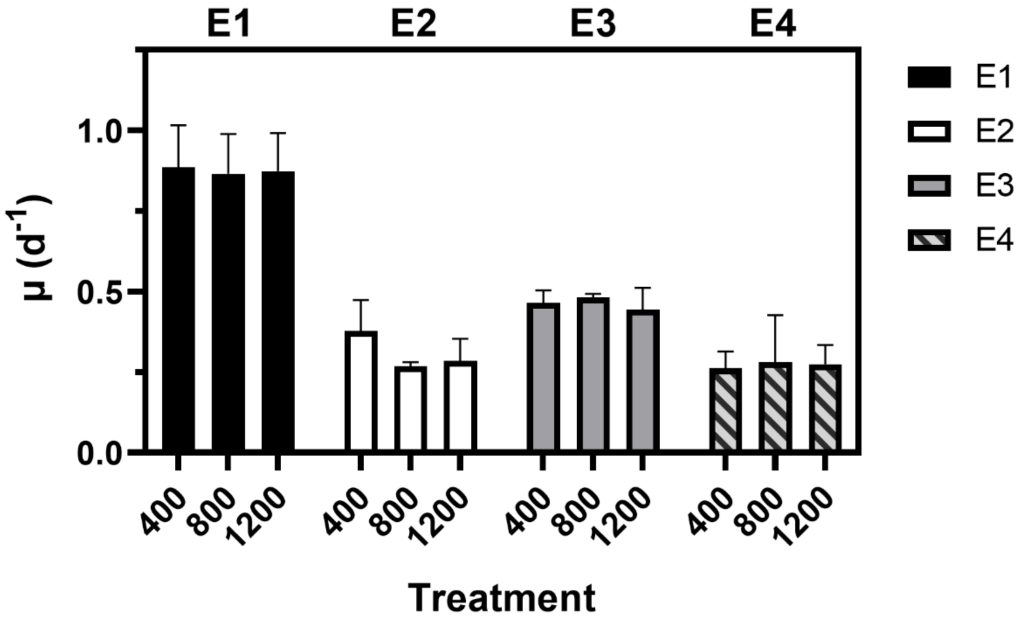

Supplementary Figure 9. The relative abundance of diatom orders and genera within diatom annotated 18S rRNA amplicons at each experiment (E1-4). Orders share colours as follows: polar centric (blue), radial centric (purple), raphid pennate (brown), araphid pennate (red), and unknown (green).

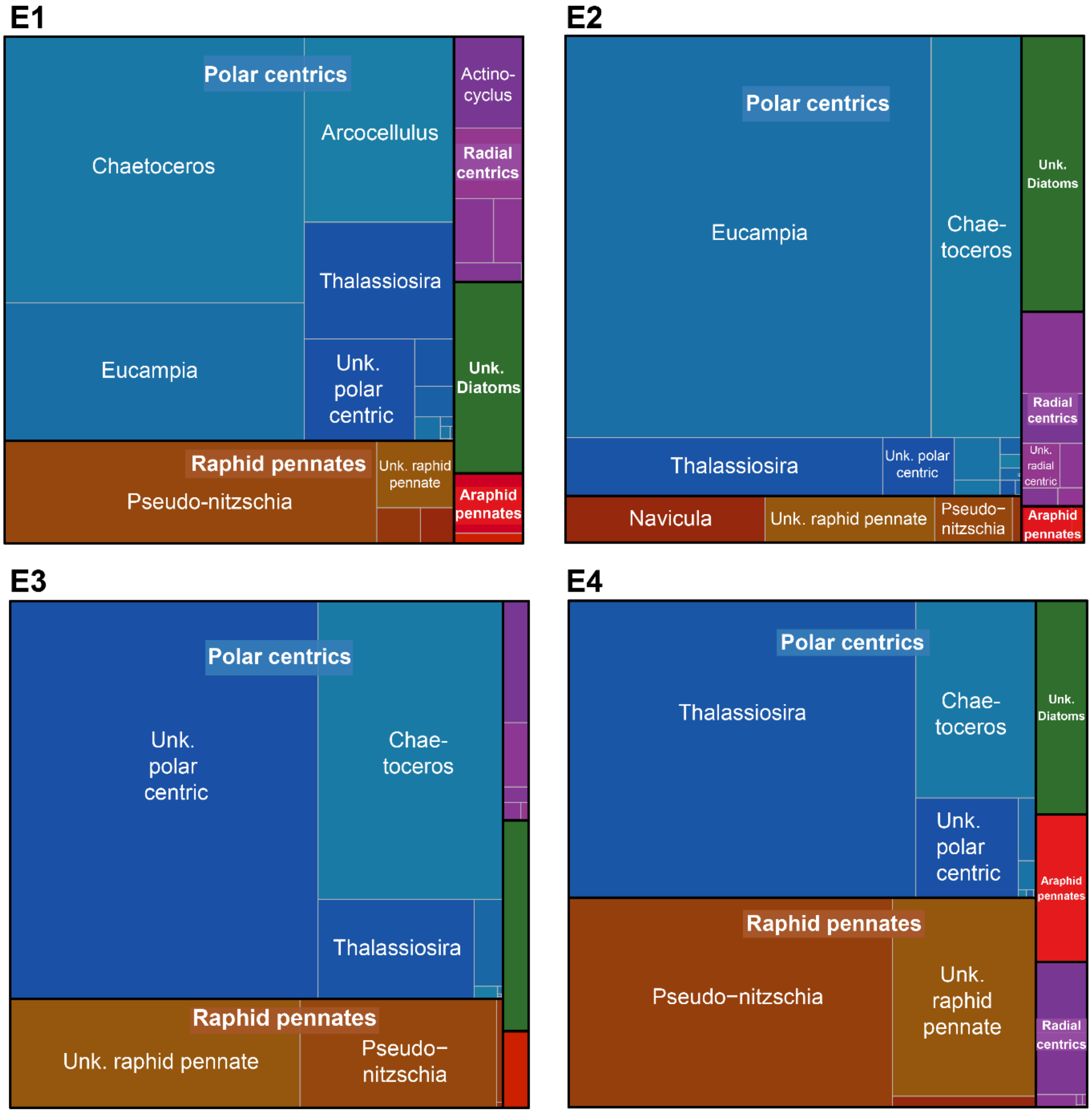

Supplementary Figure 10. The relative abundance of bacterial classes and orders from 16S rRNA amplicons at each experiment (E1-4).

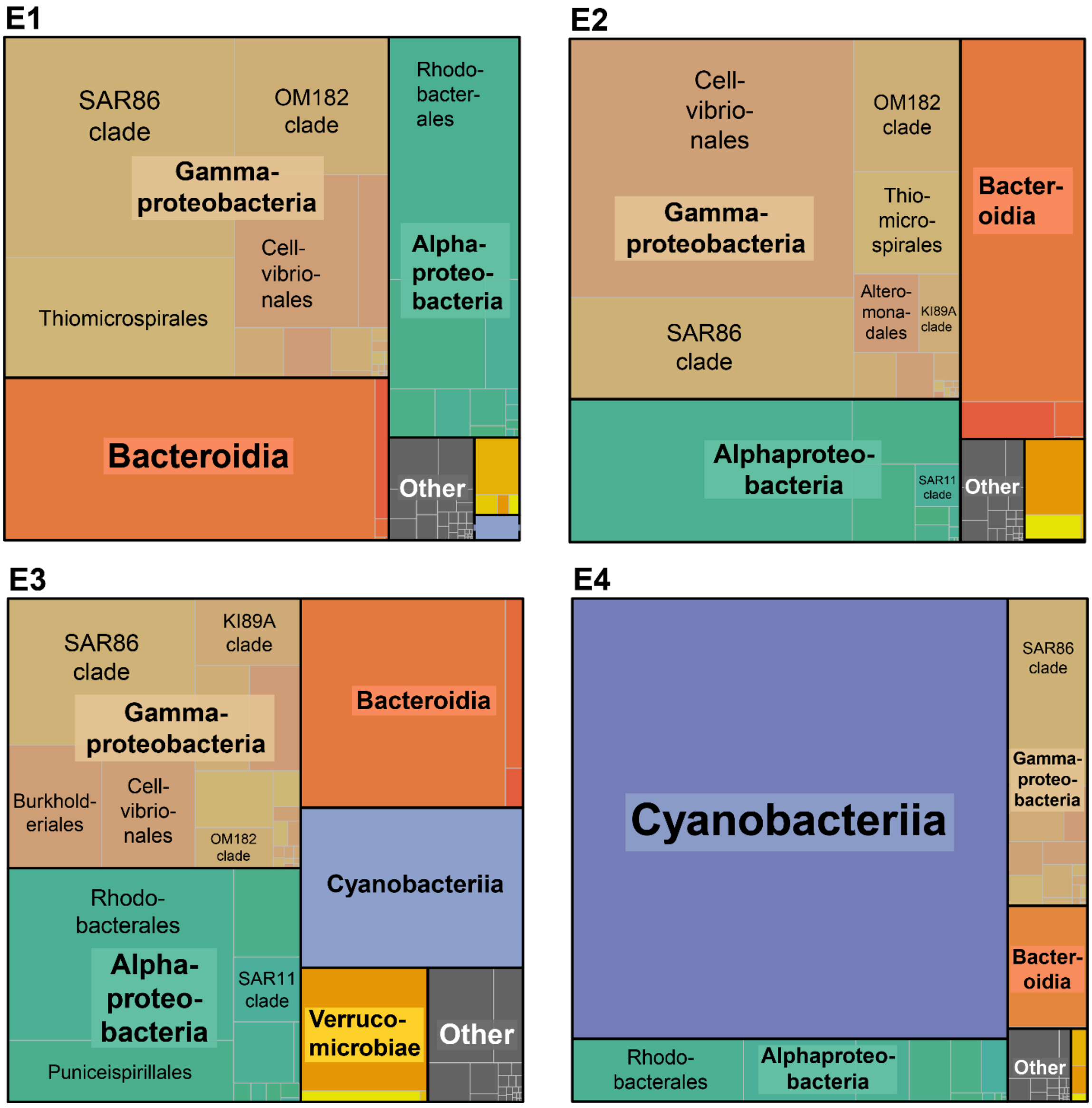

Supplementary Figure 11. PCoA plots of Bray-Curtis dissimilarity for the eukaryotic community (18S rRNA, top) and prokaryotic community (16S rRNA, bottom). Experiments and time points are denoted by colour and shape respectively (see legend).

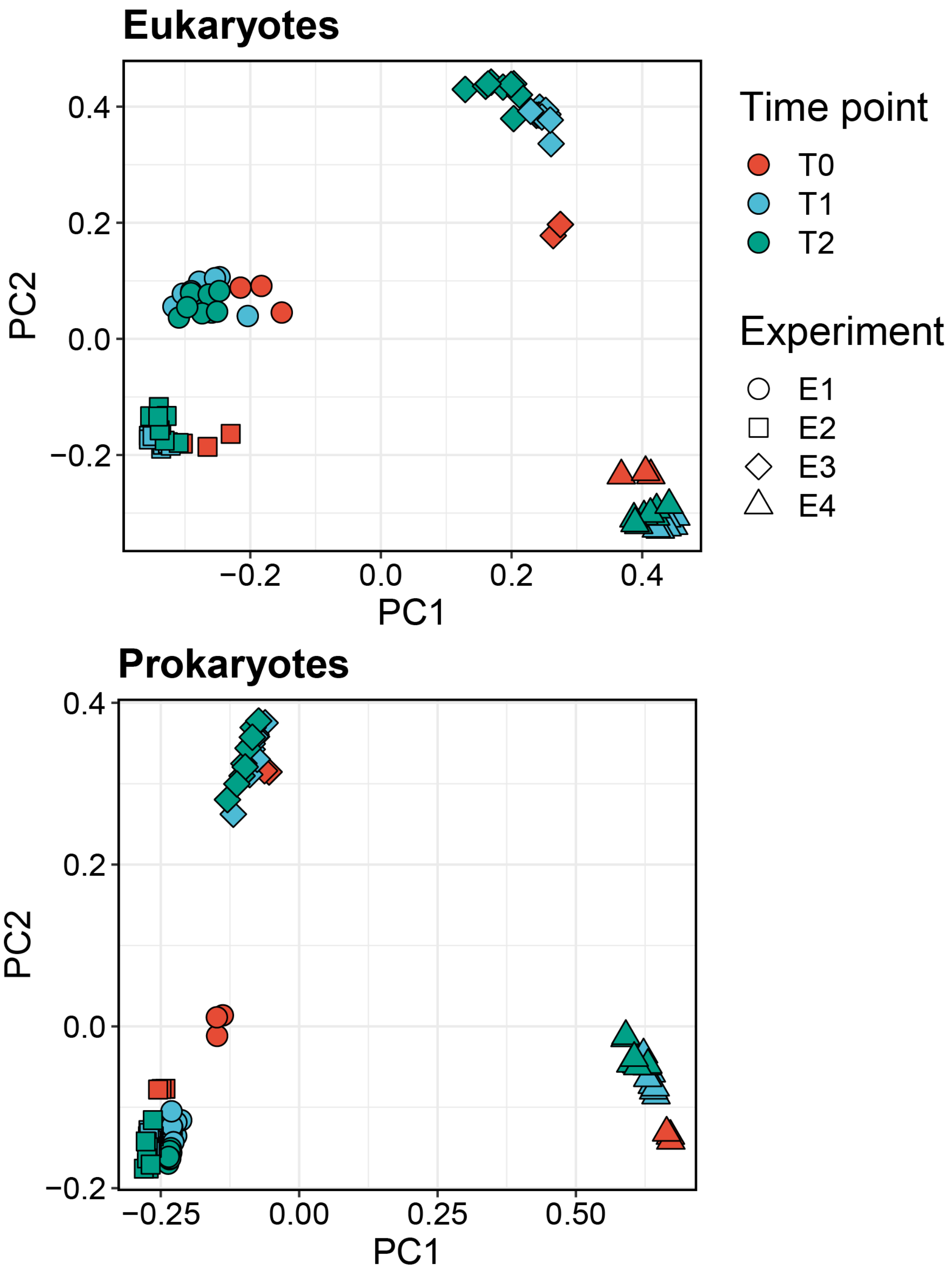

Supplementary Figure 12. The number of significantly differential abundant amplicon sequence variants (ASVs) at each experiment determined by DESeq2 between the 800 µatm (right) and 400 µatm (left) treatments at the final time point for each experiment ($P < 0.05$). Eukaryotes are shown on top and prokaryotes are shown on the bottom plot. The total number of ASVs for each experiment is shown in parentheses next to the experiment number of the y-axis.

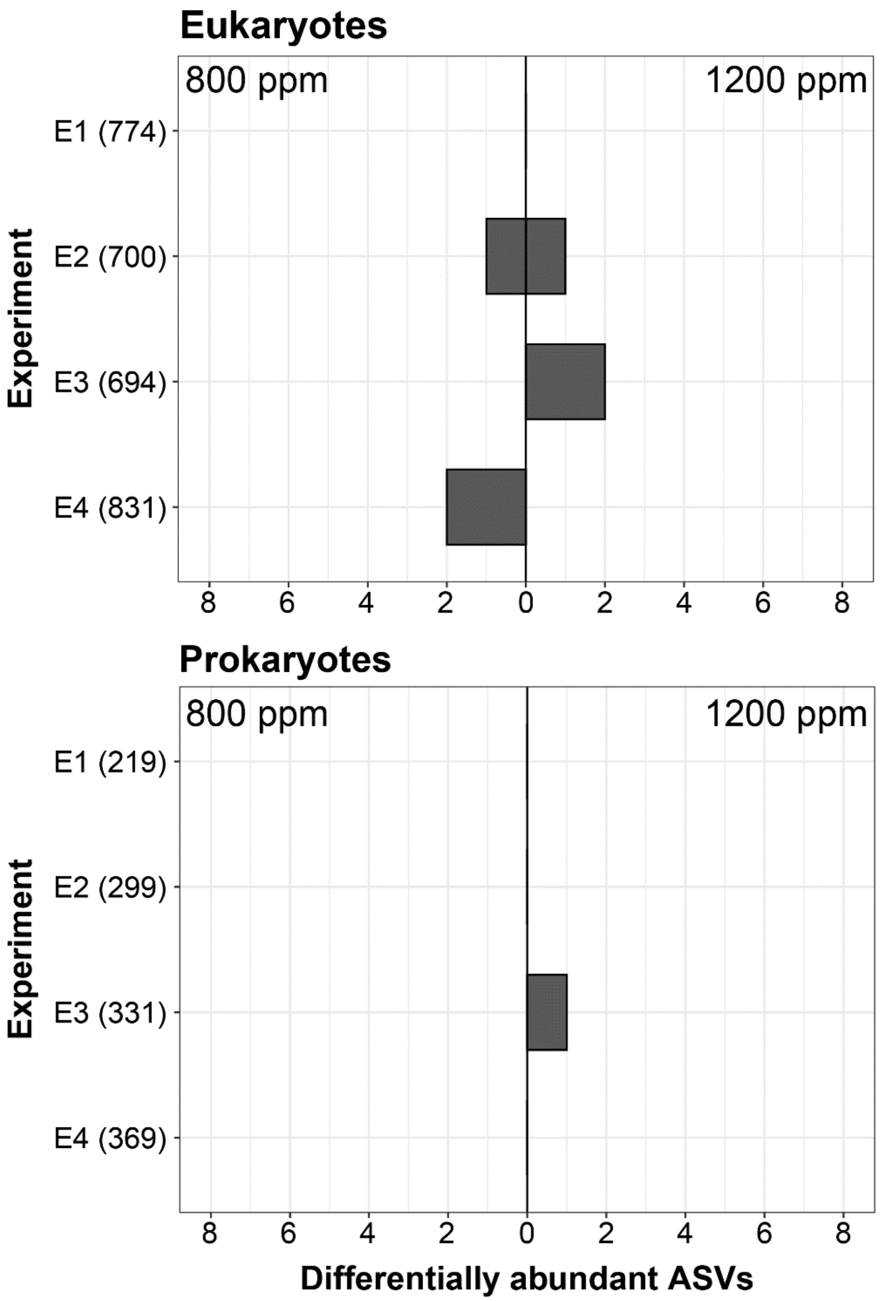

Supplementary Fig. 13. Venn diagrams of detected orthologous genes among the communities at the different experiments (E1-E4) for (A) centric diatoms and (B) pennate diatoms. The percentage of orthologous genes unique to each experiment is shown.

A. Centric Diatoms

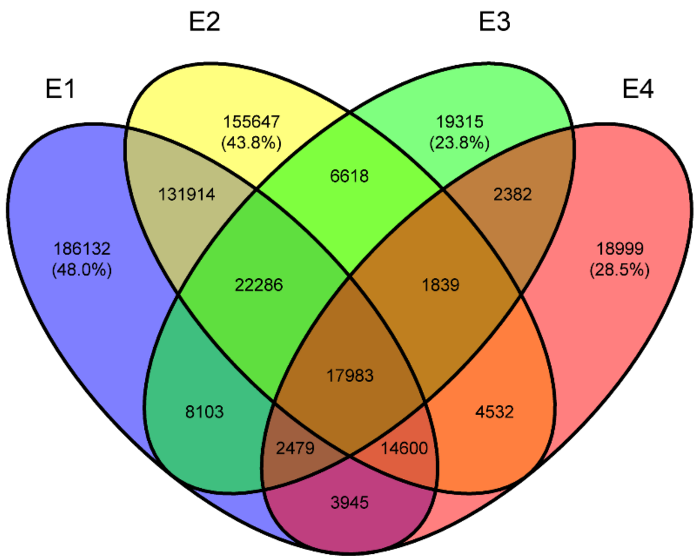

B. Pennate Diatoms

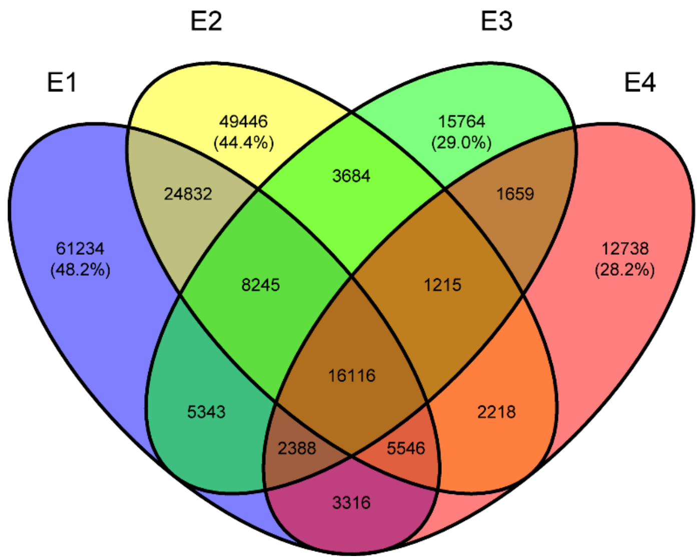

Supplementary Figure 14. Normalized transcript expression of iron starvation-induced proteins (ISIPs) in pennate diatoms (log_{10} scale): ISIP1 (red), ISIP2A (phytotransferrin or pTF, blue), ISIP3 (green). The horizontal dashed lines indicate the averaged 95th and 99th percentile for transcript abundance in each experiment. Error bars represent the standard deviation of the mean (n = 3 except the Experiment 3 T0 and Experiment 4 800 ppm samples where n = 2).

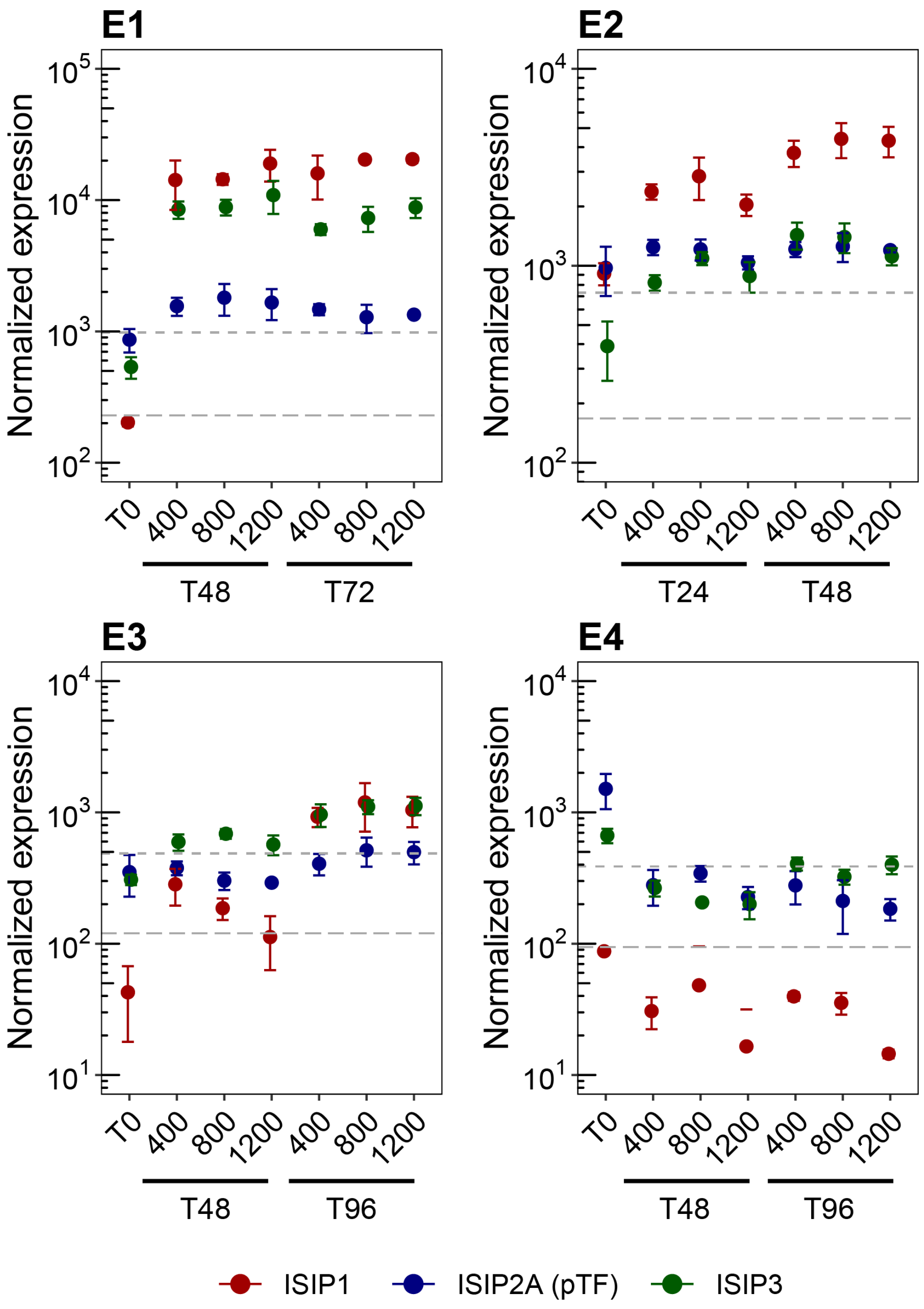

Supplementary Figure 15. Normalized (total sum scaled) protein abundances of iron starvation-induced proteins (ISIPs) in centric diatoms (top row) and pennate diatoms (bottom row). The horizontal dashed lines indicate the averaged 95th and 99th percentile for protein abundances in each experiment. Error bars represent the standard deviation of the mean (n = 3 except the Experiment 3 T0 samples where n = 2).

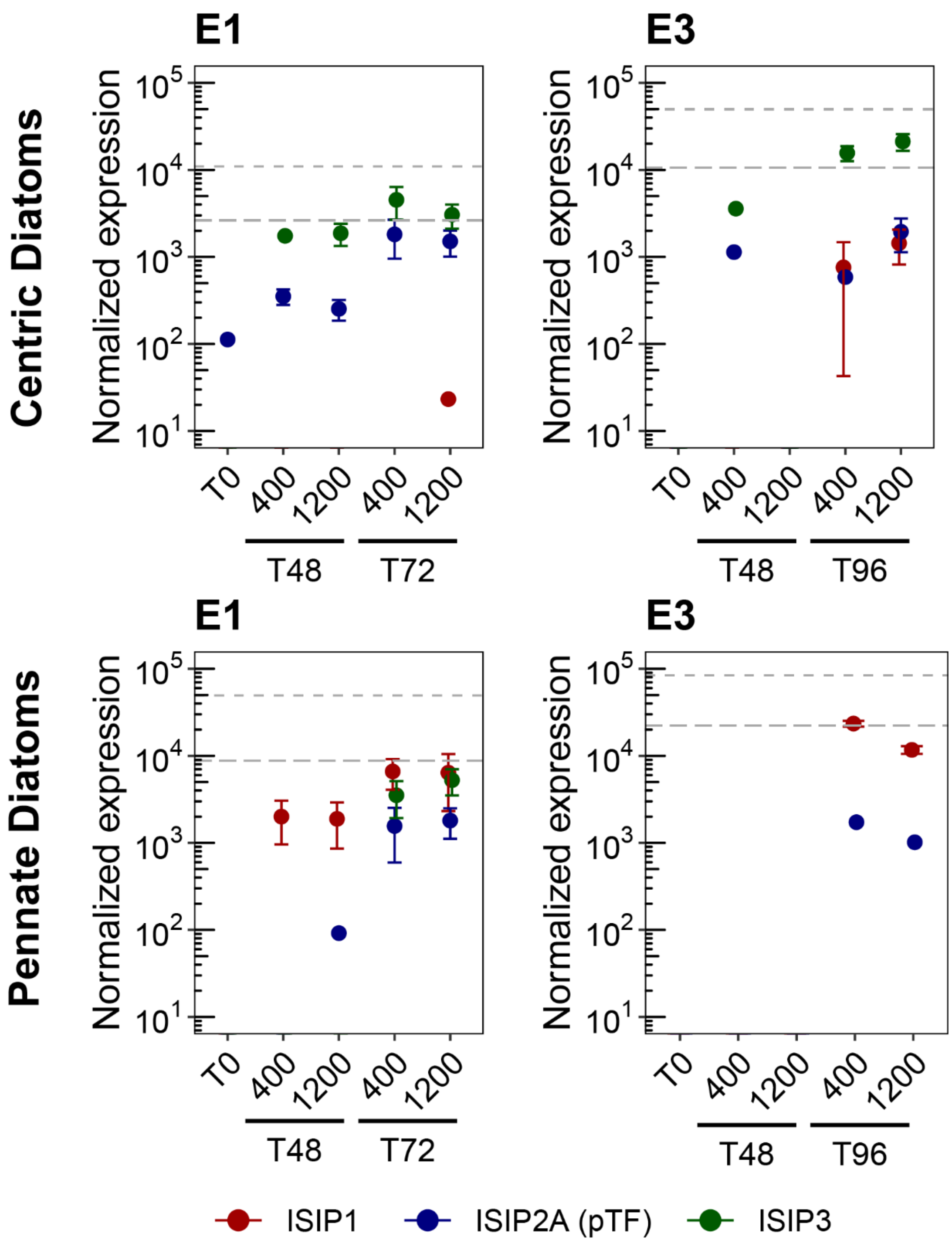

Supplementary Figure 16. Photosynthetic and ribosomal protein mass fractions for each Experiment (E), and taxonomic group with at least 50 unique peptides for each functional category. Time points (h) are denoted by shape and CO_2 treatments are denoted by colour as shown in the legend. Note that the scale of each axis is different.

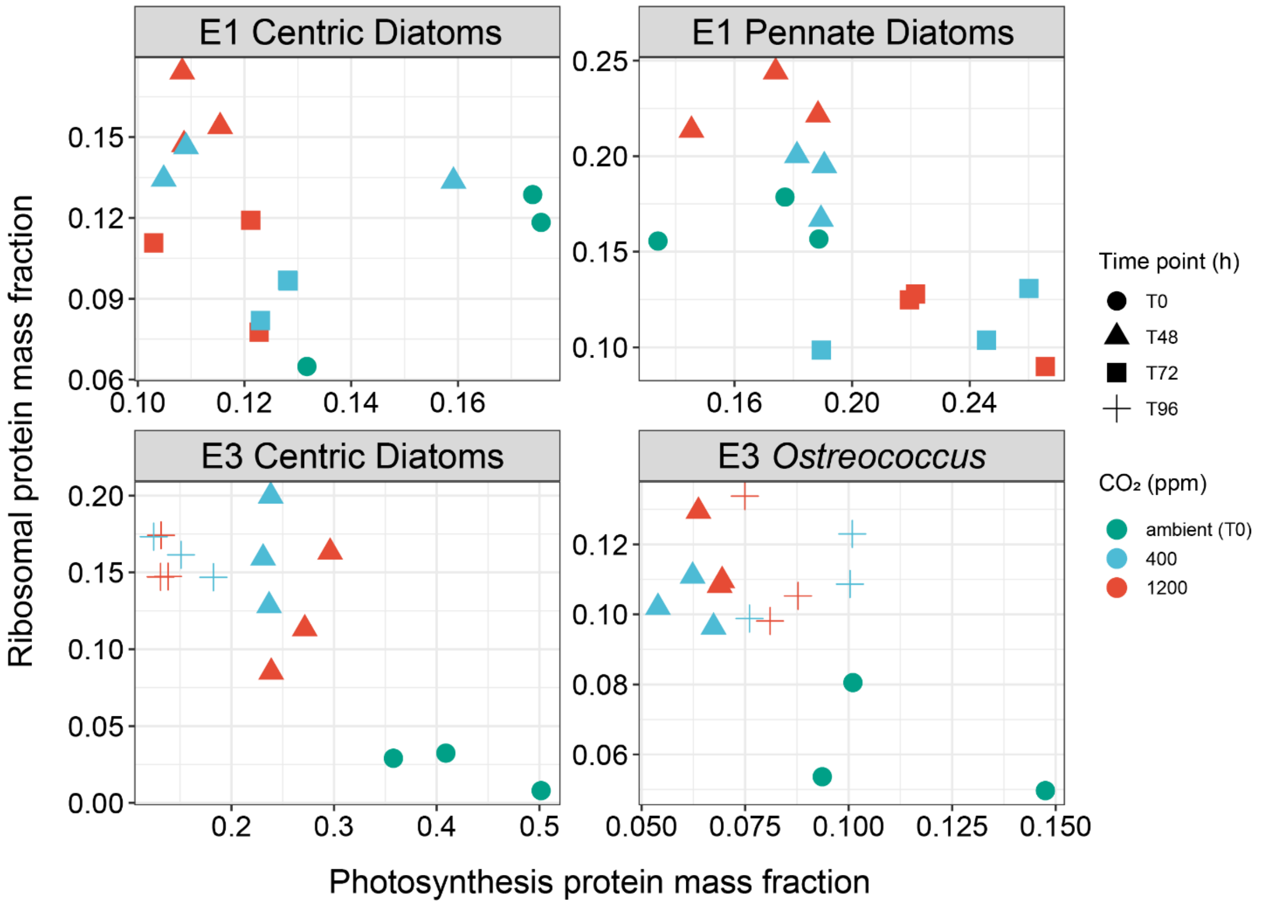

Supplementary Figure 17. Maximum likelihood phylogenetic tree as inferred from iron permease (FTR1) amino acid sequences (255 taxa/241 positions) under the LG+C40+F+R8 model as implemented in IQTree 2. The scale bar represents 0.1 amino acid substitutions per position. Branch colours and labels correspond to different taxonomic groups. Numbers above branches indicate ultra-fast Maximum-likelihood bootstrap support. A PDF version of this tree is available in this manuscript's associated figshare repository: doi.org/10.6084/m9.figshare.22589218

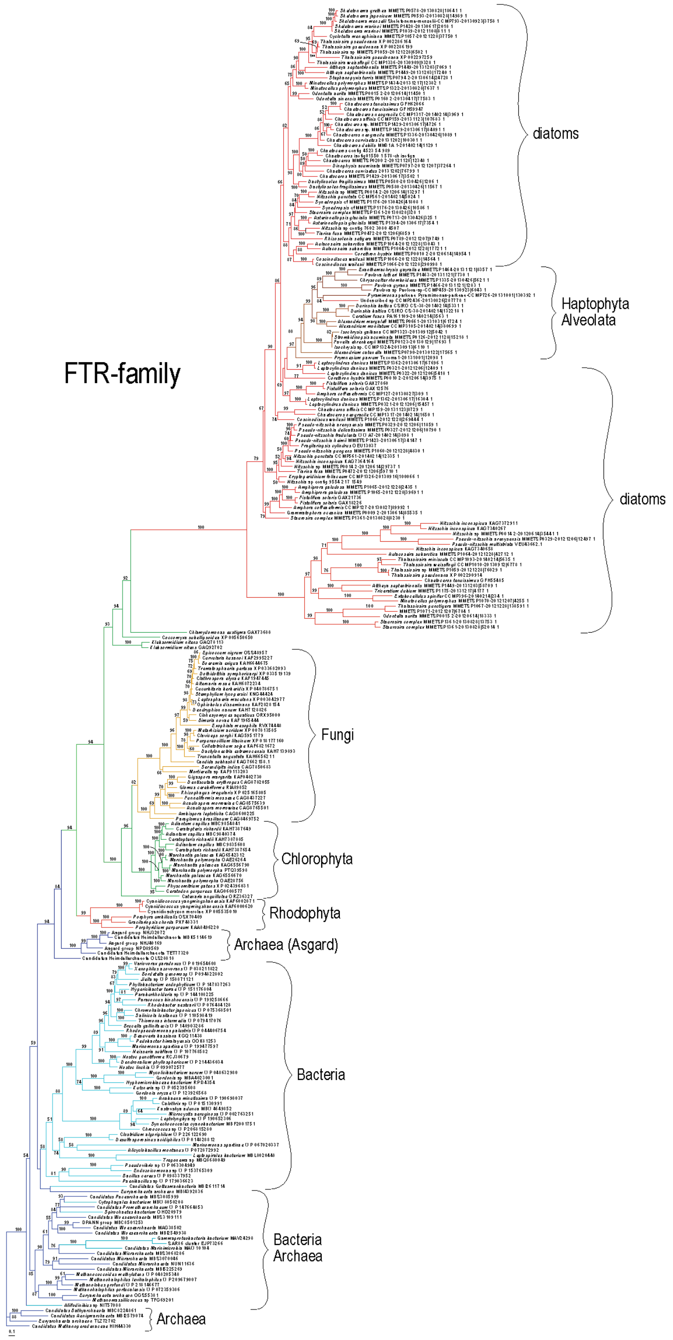

Supplementary Figure 18. Maximum likelihood phylogenetic tree as inferred from CDF-family amino acid sequences (542 taxa/309 positions) under the LG+F+R10 model as implemented in IQTree 2. The scale bar represents 0.1 amino acid substitutions per position. Branch colours and labels correspond to different taxonomic groups. Numbers above branches indicate Maximum likelihood ultra-fast bootstrap support. A PDF version of this tree is available in this manuscript's associated figshare repository: doi.org/10.6084/m9.figshare.22589218

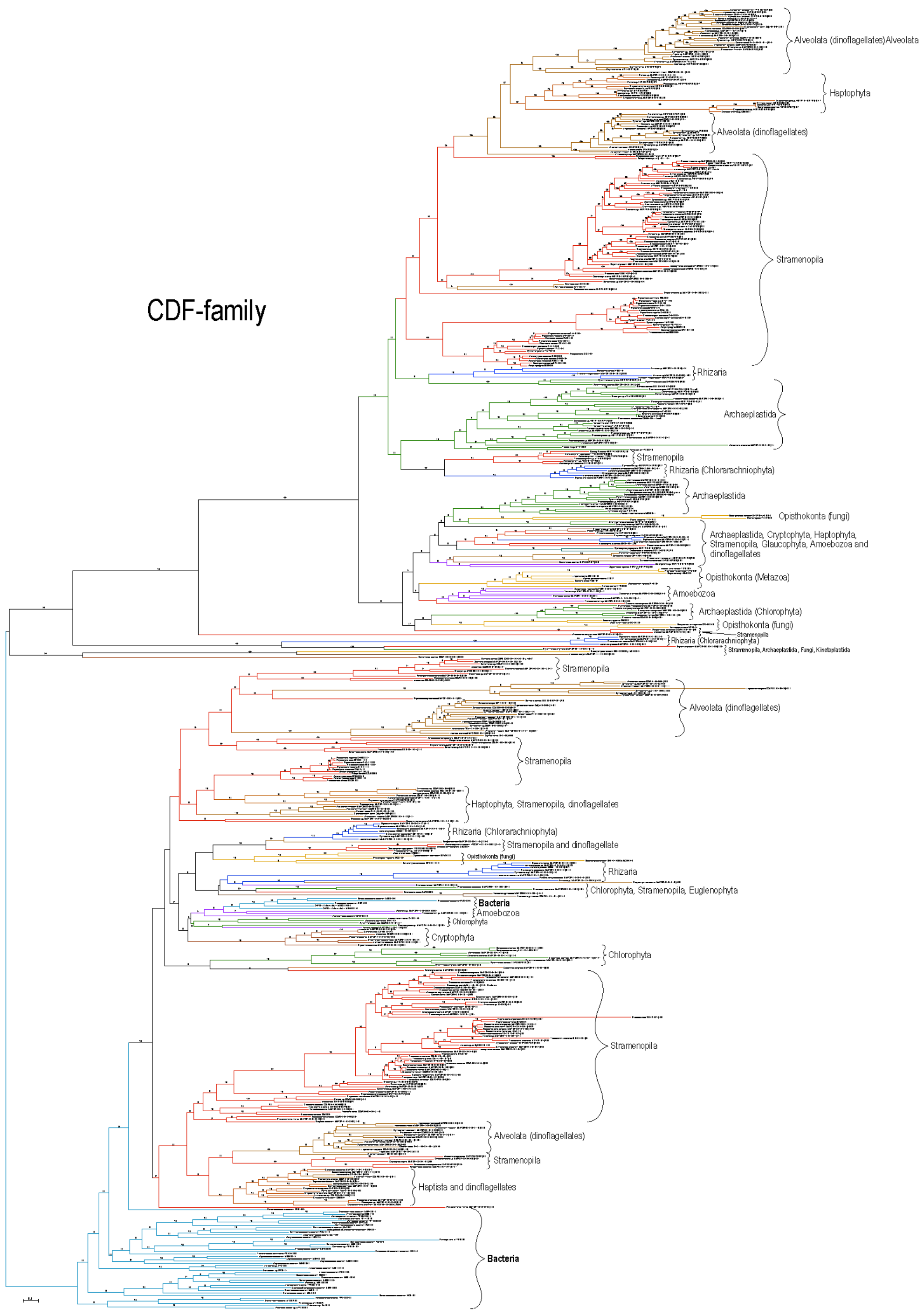

Supplementary Figure 19. Maximum likelihood phylogenetic tree as inferred from FecCD-like sequences (58 taxa/250 positions) under the LG+C20+F+R5 model as implemented in IQTree 2. The scale bar represents 0.1 amino acid substitutions per position. Branch colours and labels correspond to different taxonomic groups. Numbers above branches indicate Maximum likelihood ultra-fast bootstrap support. A PDF version of this tree is available in this manuscript's associated figshare repository: doi.org/10.6084/m9.figshare.22589218

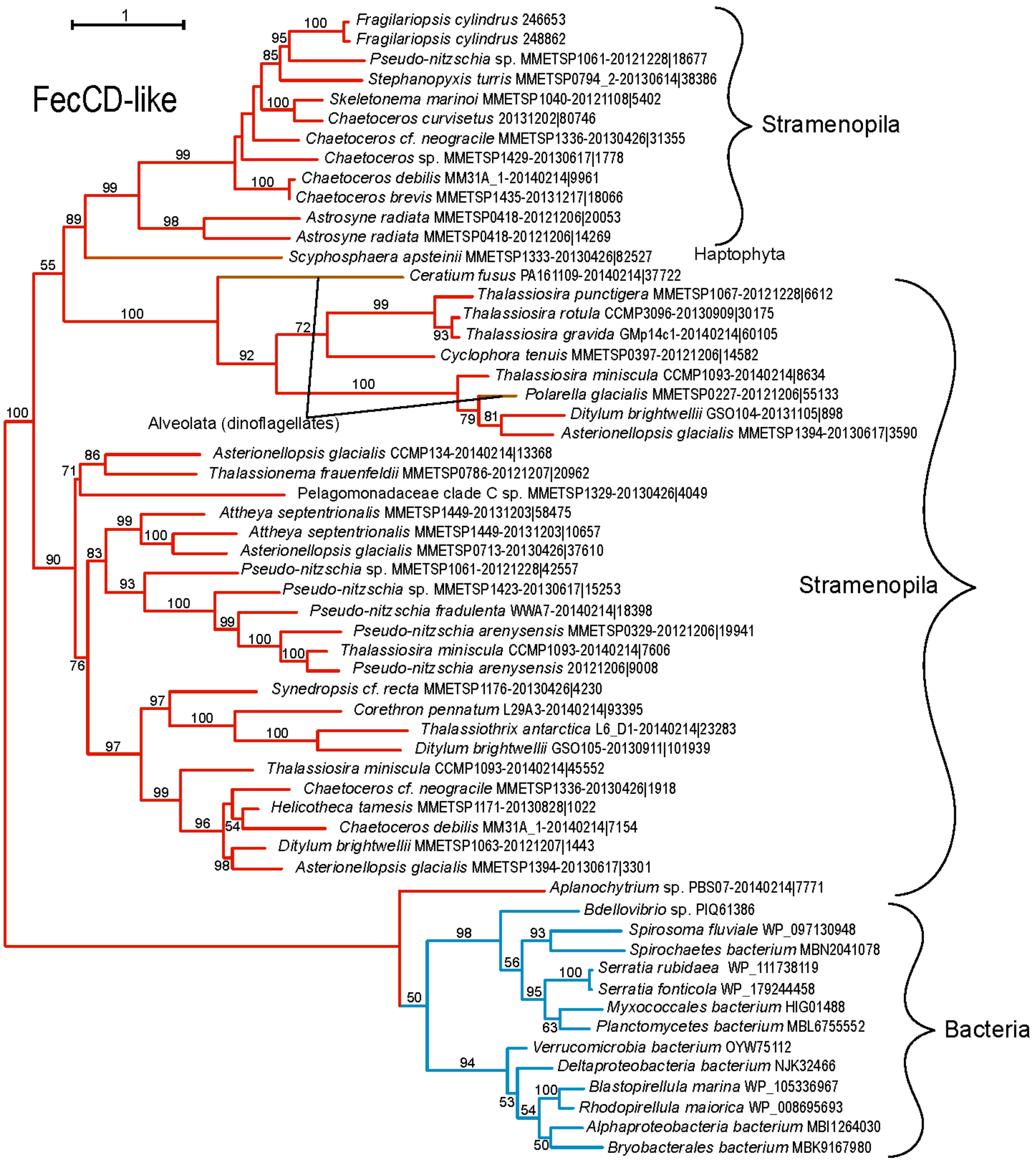

Supplementary Figure 20. Maximum likelihood phylogenetic tree as inferred from SLC49 - family amino acid sequences (374 taxa/386 positions) under the LG+C40+F+R10 model as implemented in IQTree 2. The scale bar represents 0.1 amino acid substitutions per position. Branch colours and labels correspond to different taxonomic groups. Numbers above branches indicate ultra-fast Maximum-likelihood bootstrap support. A PDF version of this tree is available in this manuscript's associated figshare repository: doi.org/10.6084/m9.figshare.22589218

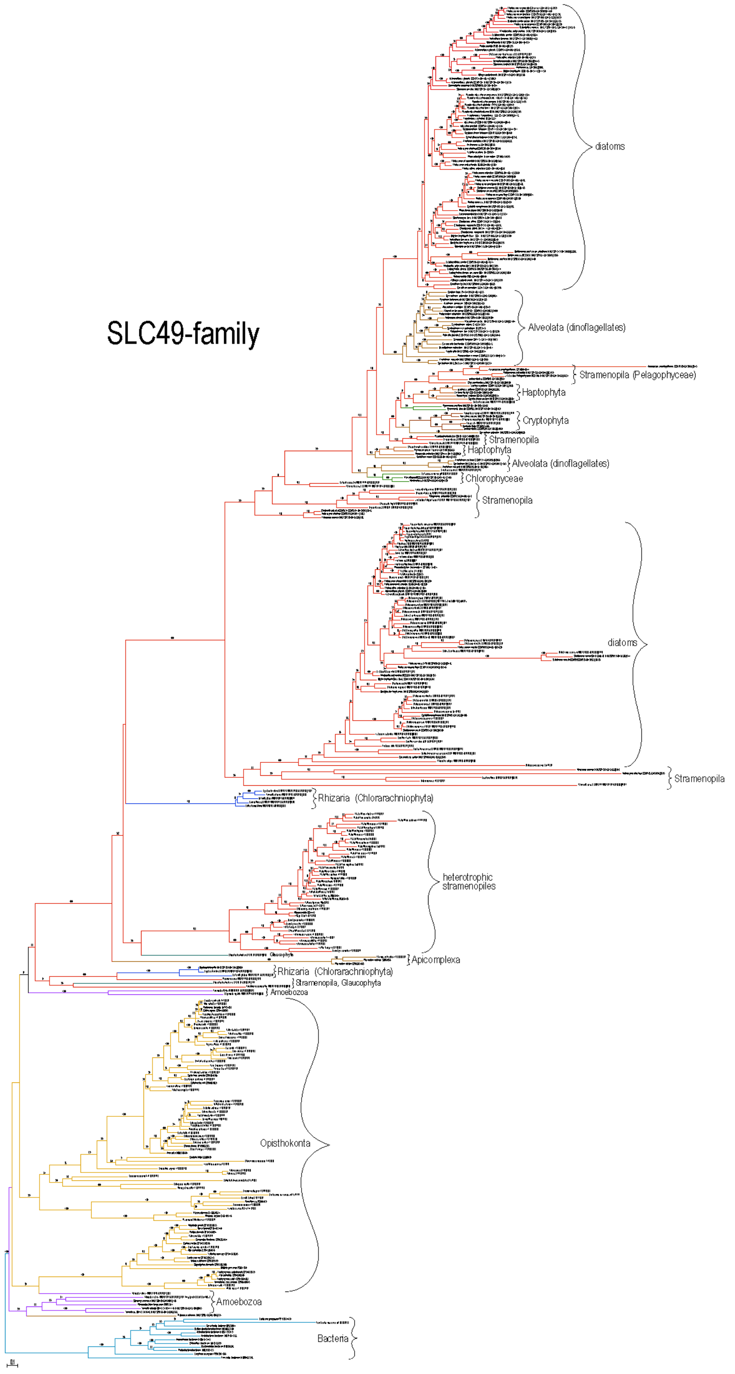

Supplementary Figure 21. Normalized transcript expression of ferrichrome binding protein 1 (FBP1) in all diatoms. Error bars represent the standard deviation of the mean (n = 3 except the Experiment 3 T0 and Experiment 4 800 ppm samples where n = 2).

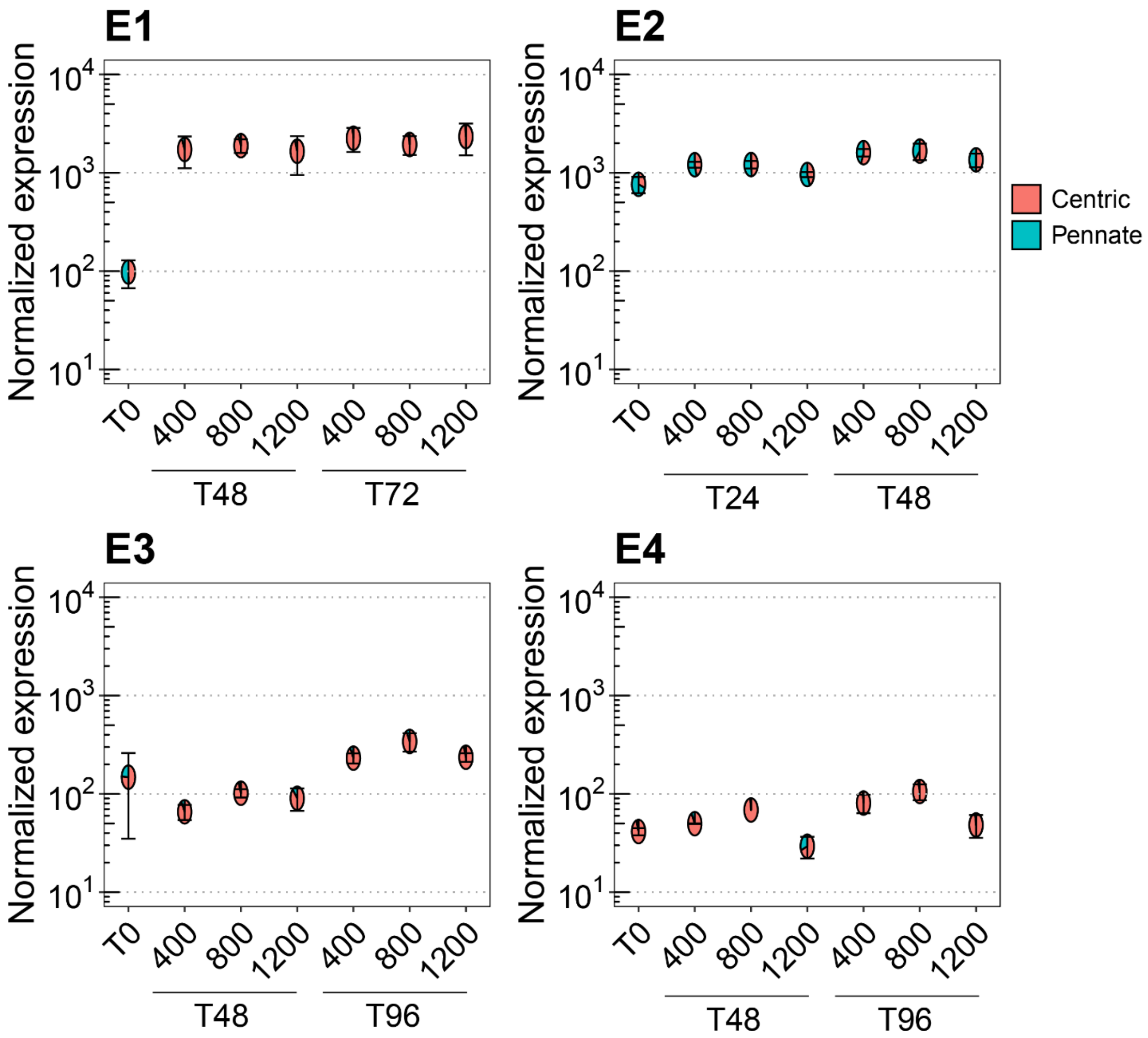

Supplementary Figure 22. Normalized (total sum scaled) protein abundances of ferrichrome binding protein 1 (FBP1) in all diatoms at Experiment 1. FBP1 proteins at E1 were either classified as unknown diatoms or centric diatoms with none assigned to pennate diatoms. FBP1 protein expression was not detected at Experiment 3. Error bars represent the standard deviation of the mean (n = 3).

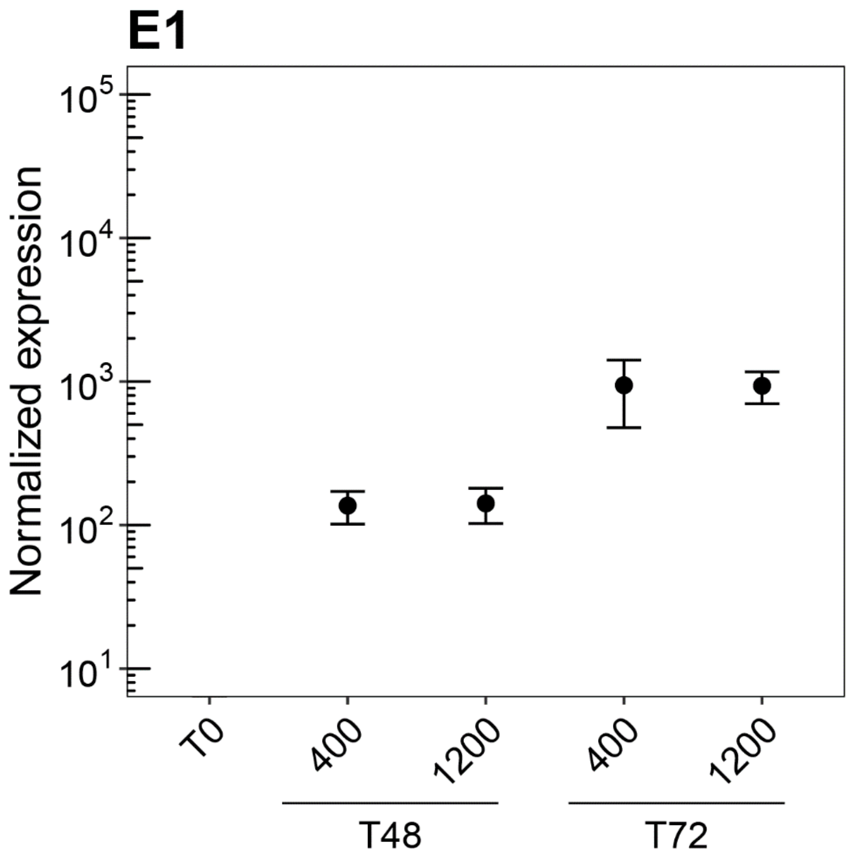

Supplementary Figure 23. MA plots for centric diatoms at Experiment 1 (T72) and Experiment 3 (T48). Comparisons are made between the 1200 ppm and 400 ppm treatments for both transcripts (top row) and proteins (bottom row). Differentially expressed genes or proteins ($P < 0.05$) are shown as pies representing the proportion of expression for dominant genera. Pie size is proportional to P-value with larger pies denoting a smaller P-value. Grey points are genes that were not significantly differentially abundance ($P > 0.05$). Data points with $\log_2$ fold changes less than -5 or greater than 5 are plotted at -5 and 5 respectively.

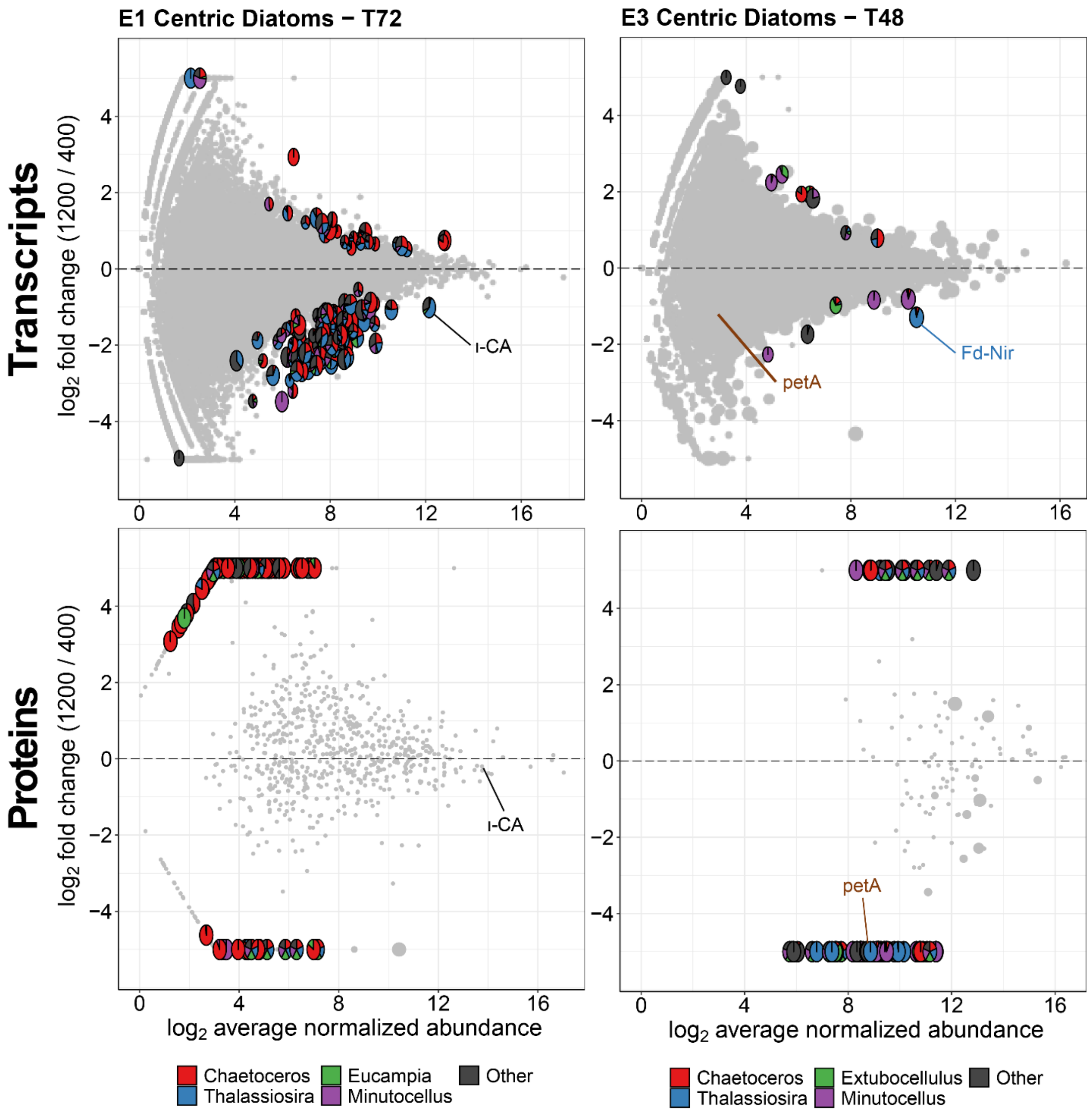

Supplementary Figure 24. Maximum likelihood phylogenetic tree as inferred from mitoNEET amino acid sequences (279 taxa/102 positions) under the LG+C40+F+R7 model as implemented in IQTree 2. The scale bar represents 1 amino acid substitutions per position. Branch colours and labels correspond to different taxonomic groups. Numbers above branches indicate ultra-fast Maximum-likelihood bootstrap support. A PDF version of this tree is available in this manuscript's associated figshare repository: doi.org/10.6084/m9.figshare.22589218

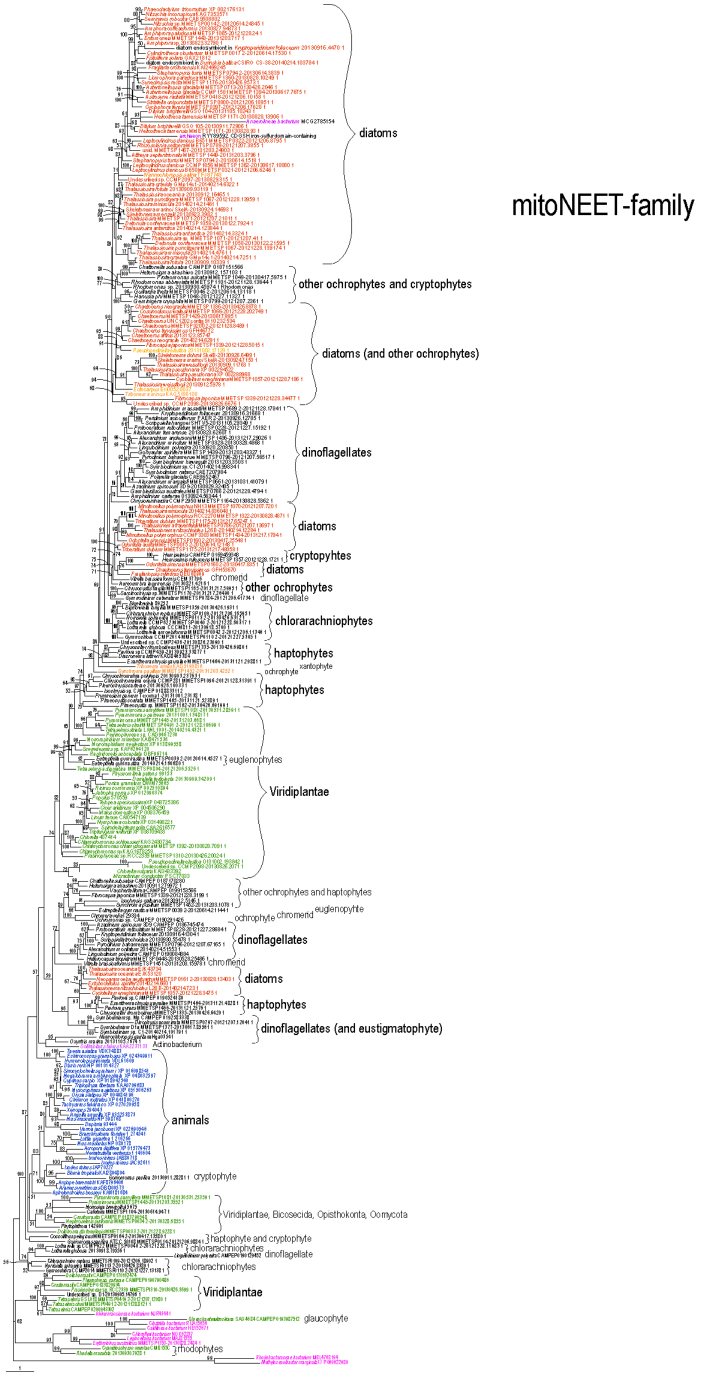

Supplementary Figure 25. Differential expression of nitrogen assimilation transcripts for certain taxonomic groups and experiments (E). Colours indicate the $\log_2$ fold change for comparisons listed above each paired time points (T). Adjusted P-values < 0.10 are shown. Genes are abbreviated as follows: NRT nitrate transporter, NAR2 nitrate transport accessory protein, NR nitrate reductase, FNT formate-nitrite transporter, Nir nitrite reductase, AMT ammonium transporter, GSII/III glutamine synthetase GOGAT, glutamate synthase.

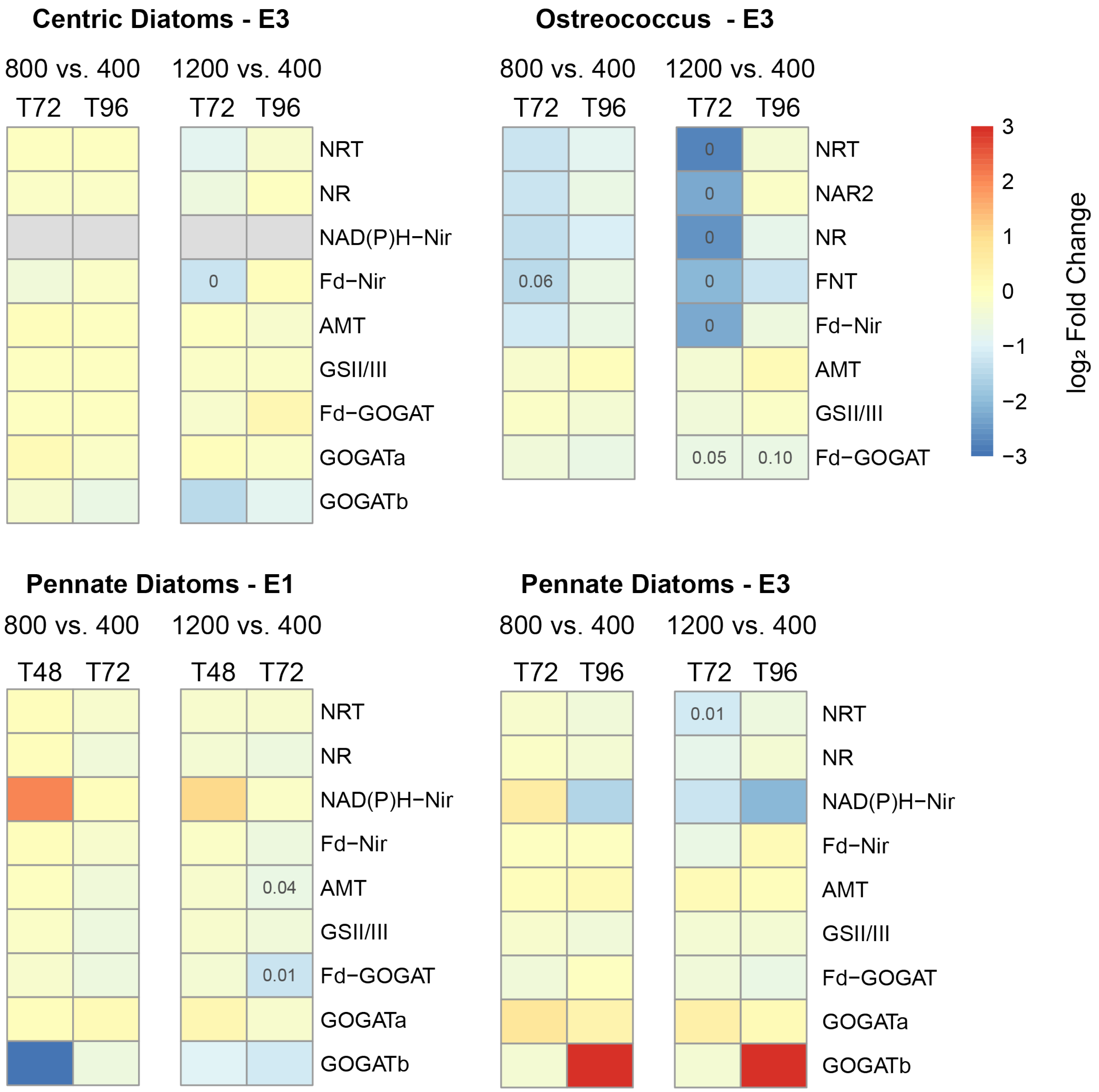

Supplementary Figure 26. MA plots for *Ostreococcus* at the second time point (96 hours) at Experiment 3. Black points are shown for significantly differentially expressed transcripts or proteins ($P < 0.05$) and are proportional to the P-value. Data points with log2 fold changes less than -5 or greater than 5 are plotted at -5 and 5 respectively.

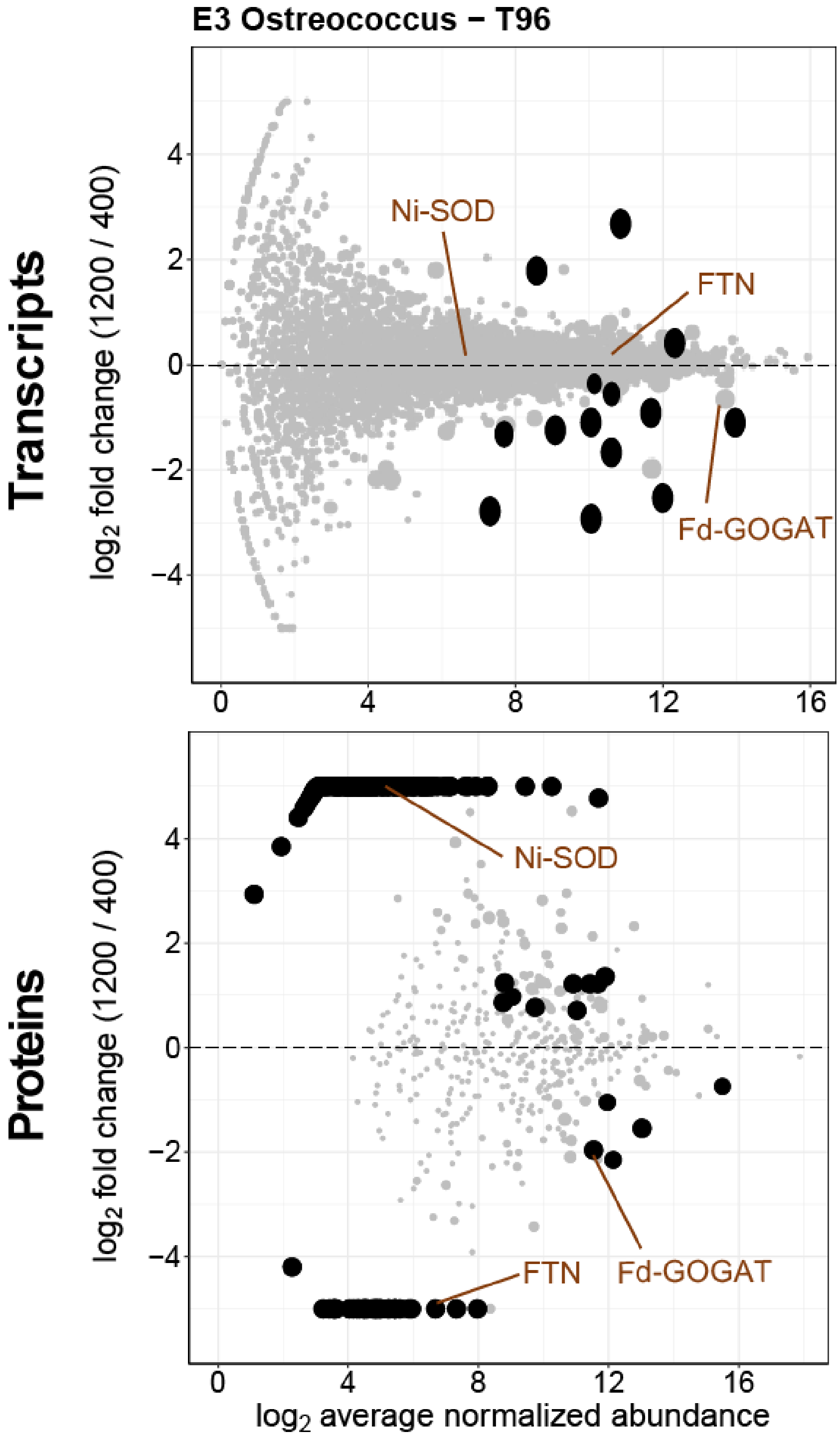

Supplementary Figure 27. SLC4-family bicarbonate transporter transcript expression ($\log_{10}$ scale) in centric diatoms (top) and pennate diatoms (bottom). The horizontal dashed lines indicate the averaged 95th and 99th percentile for transcript abundance in each experiment. Error bars indicate the standard deviation of the mean (n = 3 except the Experiment 3 T0 and Experiment 4 800 ppm samples where n = 2).

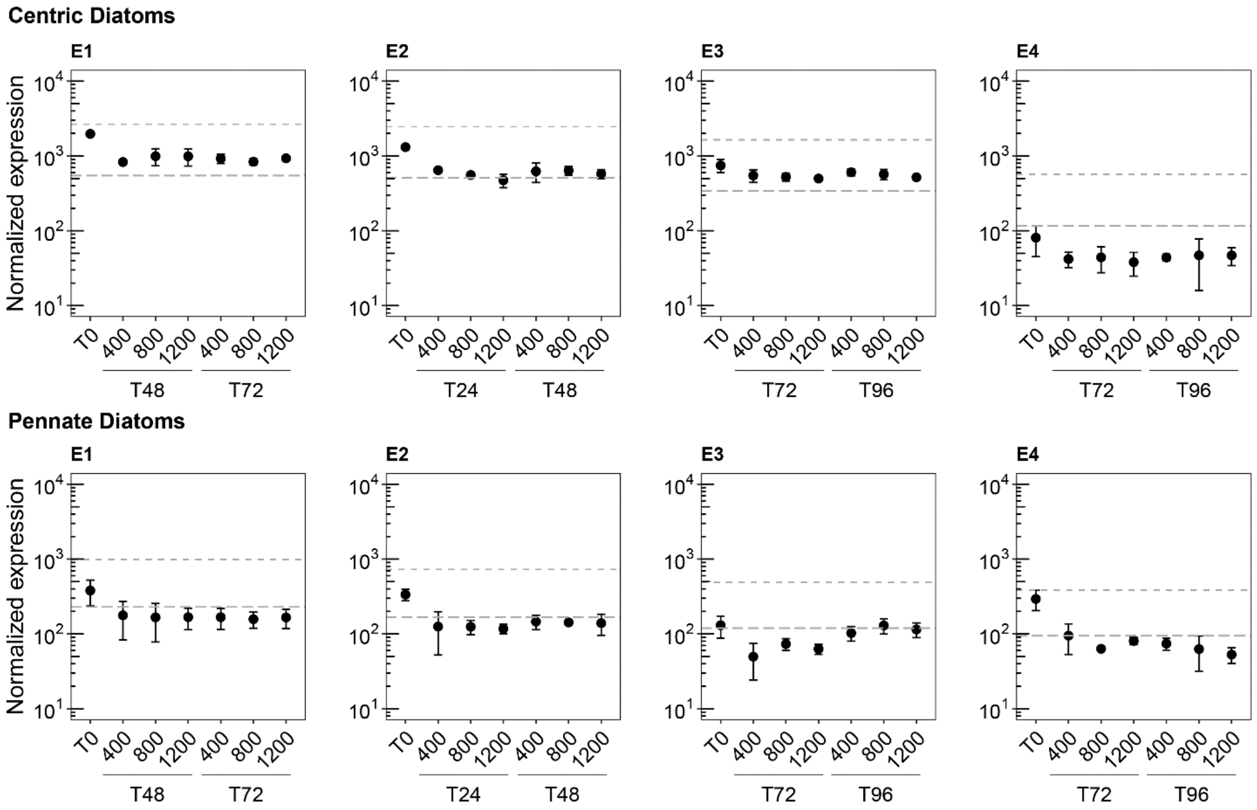

Supplementary Figure 28. Rarefaction curves for eukaryotes (18S) and prokaryotes (16S) for each sample. The vertical dashed line indicates the sampling depth for rarefaction. Colours for each sample correspond to the different experiments as shown in the legend.

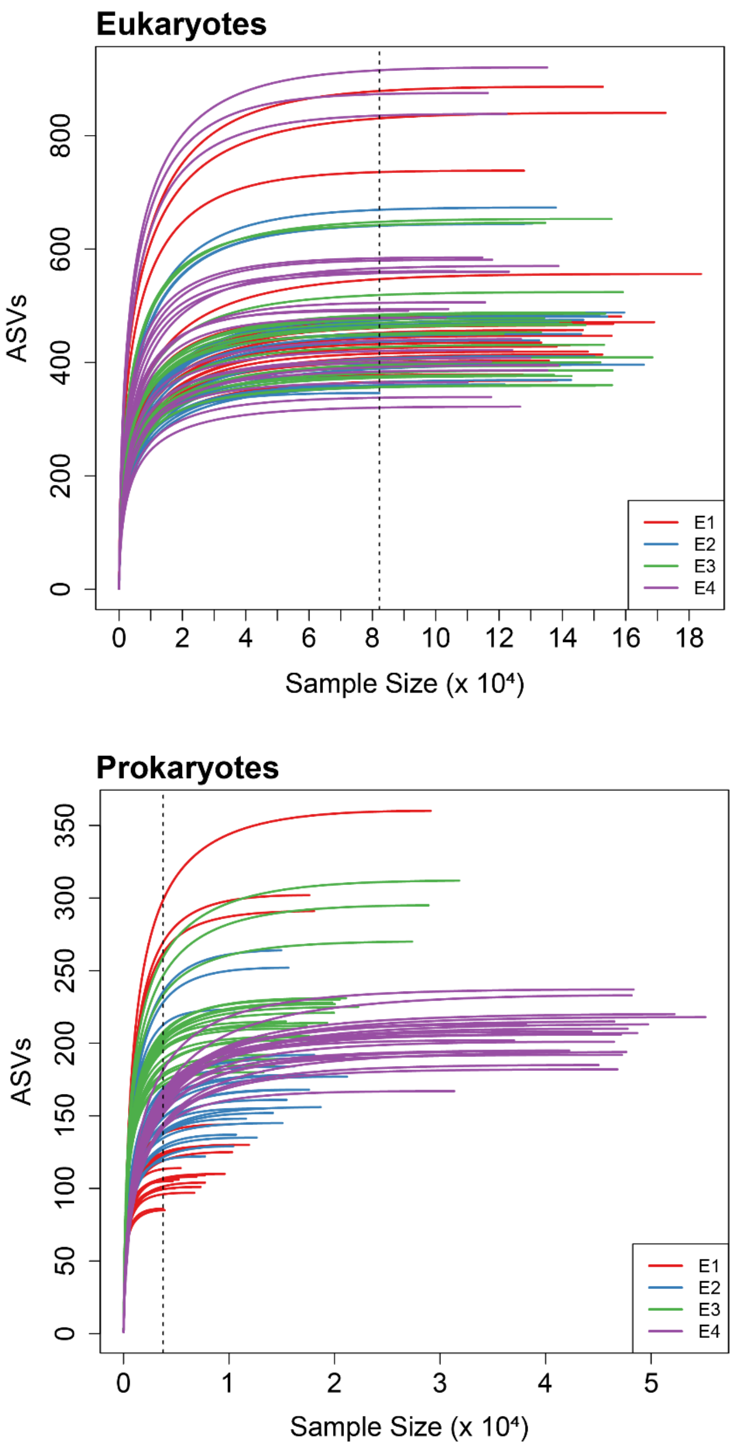

Supplementary Table 1. Reported minimum near-surface pH values in the California Current System. These pH values represent extreme cases and anomalies rather than averages with most variability existing at greater pH values. pCO_2, DIC, and CO_3 ion concentrations were estimated using seacarb[1] from pH and a constant temperature (11.8°C), salinity (32.7 psu), and total alkalinity (2218 µmol kg^{-1}) as used by Feely, et al. [2].

Data Source	Sampling or Model Years	Depth	pH	pCO_2 (µatm)	DIC (µmol kg^{-1})	CO_3 (µmol kg^{-1})
Feely et al. 2008[3]	2007	surface	7.7	944	2162	64
Hauri et al. 2013[4]	2011	surface	7.85	649	2114	88
Feely et al. 2016[5]	2007, 2011-2013		7.7	944	2162	64
Chan et al. 2017[6]	2011-2013	surface	7.43	1818	2241	36
Lilly et al. 2019[7]	2011-2018	16 m	7.64	1094	2180	56

Supplementary Table 2. Carbonate system projections for Tier 1 Shared Socioeconomic Pathway (SSP) scenarios representing the wide range of uncertainty by the year 2100[8]. Future atmospheric pCO_2 concentrations ($pCO_{2\text{-atm}}$) were obtained from the SSP Public Database Version 2.0[9]. An 800 ppm atmospheric CO_2 calculation was included as ours and previous experiments have used that concentration as a high CO_2 treatment. For all calculations, constant temperature (11.8°C) and salinity (32.7 psu) as used by Feely, et al. [2] were used. Surface seawater pCO_2 concentrations were calculated with *marelac* v2.1.10[10]. DIC, pH, and CO_3 ion concentrations were estimated using seacarb[1] from the estimated $pCO_{2\text{-sw}}$ values and a constant total alkalinity of 2234 µmol kg^{-1}, the average value from 2009-2015 CalCOFI samples in the upper 20 m.

SSP	$pCO_{2\text{-atm}}$ (ppm)	$pCO_{2\text{-sw}}$ (µatm)	DIC (µmol kg^{-1})	pH_T	CO_3 (µmol kg^{-1})
**1-2.6**	432	425	2067	8.017	125
**2-4.5**	567	557	2108	7.913	101
**N/A**	800	786	2154	7.776	76
**3-7.0**	834	820	2160	7.760	73
**5-8.5**	1089	1070	2192	7.652	58

Supplementary Table 3. Average dissolved inorganic carbon (DIC) and total alkalinity (TA) as well as calculated pH, pCO_2, and $[CO_3^{2-}]$. Errors represent the standard deviation of the mean (n = 3).

Exp.	Treat-ment	Hours	DIC (µmol kg^{-1})	TA (µmol kg^{-1})	pH_T	pCO_2 (µatm)	$[CO_3^{2-}]$ (µmol kg^{-1})
**E1**	**T0**	**0**	2123 ± 2	2247 ± 2	7.89 ± 0.00	590 ± 5	99 ± 1
	**400**	**48**	2048 ± 20	2245 ± 12	8.06 ± 0.03	382 ± 34	142 ± 9
		**72**	2063 ± 15	2254 ± 16	8.04 ± 0.02	396 ± 24	139 ± 7
	**800**	**48**	2160 ± 6	2253 ± 3	7.79 ± 0.01	763 ± 22	83 ± 2
		**72**	2171 ± 1	2264 ± 3	7.79 ± 0.01	765 ± 19	84 ± 2
	**1200**	**48**	2199 ± 8	2230 ± 6	7.59 ± 0.02	1241 ± 71	53 ± 3
		**72**	2220 ± 3	2255 ± 4	7.61 ± 0.01	1200 ± 32	56 ± 1
**E2**	**T0**	**0**	2088 ± 8	2242 ± 10	7.94 ± 0.04	516 ± 52	117 ± 11
	**400**	**24**	2082 ± 3	2262 ± 4	8.02 ± 0.00	428 ± 3	132 ± 1
		**48**	2086 ± 7	2266 ± 5	8.02 ± 0.01	427 ± 8	132 ± 1
	**800**	**24**	2180 ± 5	2264 ± 4	7.76 ± 0.02	832 ± 49	78 ± 4
		**48**	2181 ± 2	2265 ± 4	7.76 ± 0.01	830 ± 28	78 ± 2
	**1200**	**24**	2227 ± 3	2265 ± 3	7.61 ± 0.01	1192 ± 39	57 ± 2
		**48**	2230 ± 2	2268 ± 2	7.61 ± 0.01	1186 ± 36	58 ± 2
**E3**	**T0**	**0**	2022 ± 4	2244 ± 2	8.06 ± 0.01	377 ± 5	159 ± 1
	**400**	**48**	2079 ± 3	2259 ± 3	8.02 ± 0.01	425 ± 5	132 ± 1
		**96**	2085 ± 2	2272 ± 6	8.03 ± 0.01	410 ± 8	137± 3
	**800**	**48**	2176 ± 2	2261 ± 6	7.76 ± 0.01	816 ± 26	79± 3
		**96**	2181 ± 2	2274 ± 2	7.79 ± 0.00	777 ± 9	83± 1
	**1200**	**48**	2227 ± 1	2262 ± 4	7.61 ± 0.01	1208 ± 31	56± 1
		**96**	2230 ± 3	2271 ± 4	7.62 ± 0.02	1160 ± 53	59± 2
**E4**	**T0**	**0**	2016 ± 3	2221 ± 2	8.06 ± 0.01	374 ± 6	148± 2
	**400**	**48**	2062 ± 1	2242 ± 4	8.02 ± 0.01	418 ± 9	131± 2
		**96**	2067 ± 3	2245 ± 4	8.02 ± 0.00	424 ± 2	130±1
	**800**	**48**	2157 ± 4	2241 ± 7	7.77 ± 0.01	807 ± 13	77± 1
		**96**	2162 ± 5	2245 ± 4	7.76 ± 0.00	813 ± 9	77± 1
	**1200**	**48**	2207 ± 2	2241 ± 3	7.60 ± 0.00	1213 ± 5	54± 0
		**96**	2209 ± 9	2247 ± 4	7.61 ± 0.02	1179 ± 55	56± 2

Supplementary Table 4. Comparison between the number of significantly differentially expressed transcript (T) or protein (P) clusters for dominant taxonomic groups at each experiment (DESeq2 Wald Test, Benjamini & Hochberg adjusted $P < 0.05$).

Group	Exp. & Time-point	Total transcripts	Total proteins	400 ppm transcripts	400 ppm proteins	400 ppm both	1200 ppm transcripts	1200 ppm proteins	1200 ppm both
**Centric Diatoms**	**E1-T1**	27,446	926	40	57	0	155	46	1
	**E1-T2**	22,834	639	105	18	1	29	115	1
	**E3-T1**	13,603	160	6	46	0	9	15	0
	**E3-T2**	14,489	368	1	27	0	30	84	4
**Pennate diatoms**	**E1-T1**	19,518	267	0	26	0	1	26	0
	**E1-T2**	26,845	995	8	24	0	3	12	0
	**E3-T1**	12,722	89	2	27	0	0	15	0
	**E3-T2**	13,236	195	0	25	0	2	43	0
**Ostreo-coccus**	**E3-T1**	5,670	594	27	95	5	5	27	1
	**E3-T2**	5,696	595	11	40	0	3	145	0
**Pelago-monas**	**E3-T1**	7,671	83	0	16	0	0	11	0
	**E3-T2**	7,564	61	0	5	0	0	30	0

Supplementary Table 5. The number of significantly differentially expressed transcript (T) or protein (P) clusters for dominant taxonomic groups at each experiment (DESeq2 Wald Test, Benjamini & Hochberg adjusted P < 0.05). These data are presented as a heatmap in Fig. 4a.

		**800 v 400 ppm**				**1200 v 400 ppm**			
		**T1**		**T2**		**T1**		**T2**	
**Group**	**Exp.**	**400**	**800**	**400**	**800**	**400**	**1200**	**400**	**1200**
**Centric Diatoms**	**E1** (T)	1	0	2	3	40	155	105	29
	**E1** (P)	-	-	-	-	57	46	18	115
	**E2** (T)	1	7	0	0	0	0	0	0
	**E3** (T)	1	0	0	3	6	9	1	30
	**E3** (P)	-	-	-	-	46	15	27	84
	**E4** (T)	14	1	0	0	3	10	1	1
**Pennate Diatoms**	**E1 (T)**	0	1	0	0	0	1	8	3
	**E1 (P)**	-	-	-	-	26	26	24	12
	**E2 (T)**	0	1	0	0	1	4	0	1
	**E3 (T)**	0	0	0	1	2	0	0	2
	**E3 (P)**	-	-	-	-	27	15	25	43
	**E4 (T)**	0	0	0	0	0	4	0	1
**Ostreo-coccus**	**E3 (T)**	15	4	10	1	27	5	11	3
	**E3 (P)**	-	-	-	-	95	27	40	145
**Pelago-monas**	**E3 (T)**	0	0	0	0	0	0	0	0
	**E3 (P)**	-	-	-	-	16	11	5	30
	**E4 (T)**	71	25	0	0	0	0	0	0

Supplementary Table 6. Liquid chromatography flow rates and solvent composition settings. Arrows indicate linear increase or decreases in Solvent B* and A** composition.

Time (minutes)	Flow (µl/min)	% Solvent B	% Solvent A
0-15	0.3	5	95
15.1 - 90	0.25	5 → 30	95 → 70
90.1 - 102	0.25	30 → 55	70 → 45
102.1 - 106	0.3	55 → 95	45 → 5
106.1 - 110	0.3	95	5
111 - 125	0.3	5	95

*Solvent A: H_2O, 0.1% Formic Acid
** Solvent B: Acetonitrile, 0.1% Formic Acid

Supplementary Table 7. Mass spectrometer settings.

Parameter	Setting
TopN	8
Intensity Threshold	8.3e4
Dynamic Exclusion	30s
MS1 Scan Resolution	140,000
MS1 Scan Range	400 to 2000 m/z
MS1 Automatic Gain Control Target	3e6
MS2 Scan Resolution	17,500
MS2 Scan Range	200 to 2000 m/z
M2 Automatic Gain Control Target	1e6
MS2 Isolation Window	2.0 m/z

Supplementary References

1. Gattuso, J.-P. *et al.* Package 'seacarb'. (2021).
2. Feely, R. A. *et al.* The combined effects of acidification and hypoxia on pH and aragonite saturation in the coastal waters of the California current ecosystem and the northern Gulf of Mexico. *Cont Shelf Res* **152**, 50-60 (2018).
3. Feely, R. A., Sabine, C. L., Hernandez-Ayon, J. M., Ianson, D. & Hales, B. Evidence for Upwelling of Corrosive "Acidified" Water onto the Continental Shelf. *Science* **320**, 1490-1492 (2008).
4. Hauri, C. *et al.* Spatiotemporal variability and long-term trends of ocean acidification in the California Current System. *Biogeosciences* **10**, 193-216 (2013).
5. Feely, R. A. *et al.* Chemical and biological impacts of ocean acidification along the west coast of North America. *Estuar Coast Shelf Sci* **183**, 260-270 (2016).
6. Chan, F. *et al.* Persistent spatial structuring of coastal ocean acidification in the California Current System. *Sci Rep* **7**, 2526 (2017).
7. Lilly, L. E. *et al.* Biogeochemical Anomalies at Two Southern California Current System Moorings During the 2014–2016 Warm Anomaly-El Niño Sequence. *J Geophys Res Oceans* **124**, 6886-6903 (2019).
8. Gidden, M. J. *et al.* Global emissions pathways under different socioeconomic scenarios for use in CMIP6: a dataset of harmonized emissions trajectories through the end of the century. *Geosci Model Dev* **12**, 1443-1475 (2019).
9. Riahi, K. *et al.* The Shared Socioeconomic Pathways and their energy, land use, and greenhouse gas emissions implications: An overview. *Glob Environ Change* **42**, 153-168 (2017).
10. Soetaert, K., Petzoldt, T. & Meysman, F. marelac: Tools for aquatic sciences. R package version 2.1.10. (2020).
