# Supplementary material for: Short-term acidification promotes diverse iron acquisition and conservation mechanisms in upwelling-associated phytoplankton
Source: Nat Commun. 2023 Nov 8;14:7215. doi: 10.1038/s41467-023-42949-1 (PMC10632500; doi:10.1038/s41467-023-42949-1)
Supplement: Supplementary file 3 — Description of Additional Supplementary Files [file 41467_2023_42949_MOESM3_ESM.pdf]

Description of Additional Supplementary Files

File Name: Supplementary Data 1
Description: 16S ASV taxonomic assignments and raw abundances for each sample.

File Name: Supplementary Data 2
Description: 18S ASV taxonomic assignments and raw abundances for each sample.
