# Supplementary material for: Short-term acidification promotes diverse iron acquisition and conservation mechanisms in upwelling-associated phytoplankton
Source: Nat Commun. 2023 Nov 8;14:7215. doi: 10.1038/s41467-023-42949-1 (PMC10632500; doi:10.1038/s41467-023-42949-1)
Supplement: Supplementary file 6 — Reporting Summary [file 41467_2023_42949_MOESM6_ESM.pdf]

nature portfolio

Corresponding author(s): Andrew E. Allen

Last updated by author(s): Oct 10, 2023

Reporting Summary

Nature Portfolio wishes to improve the reproducibility of the work that we publish. This form provides structure for consistency and transparency in reporting. For further information on Nature Portfolio policies, see our Editorial Policies and the Editorial Policy Checklist.

Statistics

For all statistical analyses, confirm that the following items are present in the figure legend, table legend, main text, or Methods section.

n/a	Confirmed	
☐	☒	The exact sample size (*n*) for each experimental group/condition, given as a discrete number and unit of measurement
☐	☒	A statement on whether measurements were taken from distinct samples or whether the same sample was measured repeatedly
☐	☒	The statistical test(s) used AND whether they are one- or two-sided<br>*Only common tests should be described solely by name; describe more complex techniques in the Methods section.*
☒	☐	A description of all covariates tested
☐	☒	A description of any assumptions or corrections, such as tests of normality and adjustment for multiple comparisons
☐	☒	A full description of the statistical parameters including central tendency (e.g. means) or other basic estimates (e.g. regression coefficient) AND variation (e.g. standard deviation) or associated estimates of uncertainty (e.g. confidence intervals)
☐	☒	For null hypothesis testing, the test statistic (e.g. *F*, *t*, *r*) with confidence intervals, effect sizes, degrees of freedom and *P* value noted<br>*Give P values as exact values whenever suitable.*
☒	☐	For Bayesian analysis, information on the choice of priors and Markov chain Monte Carlo settings
☒	☐	For hierarchical and complex designs, identification of the appropriate level for tests and full reporting of outcomes
☒	☐	Estimates of effect sizes (e.g. Cohen's *d*, Pearson's *r*), indicating how they were calculated

Our web collection on statistics for biologists contains articles on many of the points above.

Software and code

Policy information about availability of computer code

Data collection	No software was used for data collection.
Data analysis	matplotlib v2.2.2, Python v2.7, CO2SYS for MATLAB v2.1, MATLAB, R, Microsoft Excel 2019, QIIME2 v2019.10, DESeq2 v1.22.2, FastQC v0.11.8, MultiQC v1.9dev0, Trinity v2.8.5, Trans-Abyss v2.0.1, GenemarkS-T, BBMap v38.79, SortMeRNA v2.1, DIAMOND v0.9.31, kofamscan, ThermoRawFileParser, OpenMS MSGF+, BLASTP, USEARCH, MAFFT v7, SeaView 4, IQTree 2, Graphpad PRISM v9.0.0.

For manuscripts utilizing custom algorithms or software that are central to the research but not yet described in published literature, software must be made available to editors and reviewers. We strongly encourage code deposition in a community repository (e.g. GitHub). See the Nature Portfolio guidelines for submitting code & software for further information.

Data

Policy information about availability of data

All manuscripts must include a data availability statement. This statement should provide the following information, where applicable:

- Accession codes, unique identifiers, or web links for publicly available datasets
- A description of any restrictions on data availability
- For clinical datasets or third party data, please ensure that the statement adheres to our policy

The sequence data for the metatranscriptome and rRNA amplicon libraries reported in this study have been deposited in the National Center for Biotechnology (NCBI) sequence read archive under the BioProject accession no. PRJNA787648. Metatranscriptome assembiles, read counts, and annotations are available at Zenodo (https://doi.org/10.5281/zenodo.5758778). The ASV tables with abundances and taxonomic annotations are supplied as Supplementary Datasets S1 and S2 for 18S and 16S rRNA respectively. The mass spectrometry proteomics data have been deposited to the ProteomeXchange Consortium via the PRIDE partner repository with the dataset identifier PXD038549.

Field-specific reporting

Please select the one below that is the best fit for your research. If you are not sure, read the appropriate sections before making your selection.

☐ Life sciences ☐ Behavioural & social sciences ☒ Ecological, evolutionary & environmental sciences

For a reference copy of the document with all sections, see nature.com/documents/nr-reporting-summary-flat.pdf

Ecological, evolutionary & environmental sciences study design

All studies must disclose on these points even when the disclosure is negative.

Study description	The goal of this study was to examine the effects of acidification on natural phytoplankton communities in an upwelling region while considering iron bioavailability. Natural phytoplankton communities from the California Current System were collected and incubated using trace metal clean techniques in an incubator in the ship's lab at 12.5°C with 115 µE photosynthetically active radiation. Seawater was continuously bubbled with commercially prepared gas mixtures of air balanced with 400, 800, or 1200 ppm CO2 (Praxair, Inc.) creating three treatments each with triplicate bottles.
Research sample	Natural communities from whole seawater were collected to study organisms from the region.
Sampling strategy	Water was selected based on the availability of ship time, resources on board, and the depth of maximal fluorescence. The availability of resources dictated the sample size (n = 3). Seawater was collected directly from the Niskin bottles and immediately processed for the initial time point. For sampling later timepoints or chlorophyll a at 24-hour intervals, bottles were brought into a HEPA-filtered positive pressure trace metal clean area, samples were immediately taken for dissolved inorganic carbon (DIC), and then subsamples for chlorophyll a, particulate organic carbon and nitrogen, RNA, proteins, Fe uptake, macronutrient concentrations, and total alkalinity (TA) were dispensed into additional bottles for sampling.
Data collection	Data were recorded in the ship's logger and at the time of collection on log sheets by those performing the experiments.
Timing and spatial scale	Experiments were conducted during 2019: August 13th, August 19th, August 24th, and August 30th. The intervals were determined based on time to complete each experiment and availability of time and materials on board the research vessel.
Data exclusions	One replicate from the 800 ppm treatment for experiment 4 (800B) was not included in final analyses due to potential iron contamination.
Reproducibility	All experiments were conducted in the field using natural marine communities. As such, it would not be possible to repeat the experiment with the exact same community composition and conditions. Detailed methods of the experimental design and sampling are provided to ensure reproducibility of the approaches used.
Randomization	Bottles were randomly allocated into treatment groups.
Blinding	Except for hydrographic and fluorometric data from the CTD rosette, there was no prior knowledge of the initial conditions. Analysis was performed by those who designed and/or conducted the experiment and was not blinded.

Did the study involve field work? ☒ Yes ☐ No

Field work, collection and transport

Field conditions	Sampling was conducted in the California Current System during summer (August 2020) with upwelling conditions typical for the region during the time of year.
Location	Four experiments were conducted in the California Current System. Experiment 1 was conducted at 36° 7.398 N 122° 2.475 W with initial seawater collection from 12 meters. Experiment 2 was conducted at 36° 7.333 N 122° 40.441 W with seawater collected from 15 meters. Experiment 3 was conducted at 35° 34.823 N 122° 39.551 W with seawater collected from 16 meters. Experiment 4 was conducted at 34° 53.818 N 124° 46.905 W with seawater collected from 60 meters.
Access & import/export	All sampling was conducted on-board the R/V Atlantis in compliance with all relevant laws. Compliance was overseen by the Captain and crew of the vessel.
Disturbance	No disturbances were caused by this study.

Reporting for specific materials, systems and methods

We require information from authors about some types of materials, experimental systems and methods used in many studies. Here, indicate whether each material, system or method listed is relevant to your study. If you are not sure if a list item applies to your research, read the appropriate section before selecting a response.

Materials & experimental systems

n/a	Involved in the study
☒	☐ Antibodies
☒	☐ Eukaryotic cell lines
☒	☐ Palaeontology and archaeology
☒	☐ Animals and other organisms
☒	☐ Human research participants
☒	☐ Clinical data
☒	☐ Dual use research of concern

Methods

n/a	Involved in the study
☒	☐ ChIP-seq
☒	☐ Flow cytometry
☒	☐ MRI-based neuroimaging
